# 1-Pyrroline-5-carboxylate released by prostate Cancer cell inhibit T cell proliferation and function by targeting SHP1/cytochrome c oxidoreductase/ROS Axis

**DOI:** 10.1186/s40425-018-0466-z

**Published:** 2018-12-13

**Authors:** Yutao Yan, Lei Chang, Hongzhe Tian, Lu Wang, Yawei Zhang, Tao Yang, Guohao Li, Weifeng Hu, Kavita Shah, Gang Chen, Yonglian Guo

**Affiliations:** 1 Department of Urology, Central Hospital of Wuhan, Tongji Medical College, Huazhong University of Science and Technology, Wuhan, China; 2 Institute of Organ Transplantation, Tongji Hospital, Tongji Medical College, Huazhong University of Science and Technology, Wuhan, China; 3 Key Laboratory of Organ Transplantation, Ministry of Health, Wuhan, China; 4 Key Laboratory of Organ Transplantation, Ministry of Education, Wuhan, China; 5 Department of Organ Transplantation, Changhai Hospital, Second Military Medical University, Shanghai, China; 6 Department of Urology, Wuhan Children’s Hospital (Wuhan Maternal and Child Healthcare Hospital), Tongji Medical College, Huazhong University of Science and Technology, Wuhan, China; 7 Department of Urology, Jingzhou Central Hospital, the Second Clinical Medical College, Yangtze University, Jingzhou, China; 8 Department of Chemistry and Purdue University Center for Cancer Research, Purdue University, West Lafayette, IN USA

**Keywords:** 1-Pyrroline-5-carboxylate, T cell, Prostate cancer, SHP1, ROS

## Abstract

**Background:**

Tumor cell mediated immune-suppression remains a question of interest in tumor biology. In this study, we focused on the metabolites that are released by prostate cancer cells (PCC), which could potentially attenuate T cell immunity.

**Methods:**

Prostate cancer cells (PCC) media (PCM) was used to treat T cells, and its impact on T cell signaling was evaluated. The molecular mechanism was further verified in vivo using mouse models. The clinical significance was determined using IHC in human clinical specimens. Liquid chromatography mass spectroscopy (LC/MS-MS) was used to identify the metabolites that are released by PCC, which trigger T cells inactivation.

**Results:**

PCM inhibits T cells proliferation and impairs their ability to produce inflammatory cytokines. PCM decreases ATP production and increases ROS production in T cells by inhibiting complex III of the electron transport chain. We further show that SHP1 as the key molecule that is upregulated in T cells in response to PCM, inhibition of which reverses the phenotype induced by PCM. Using metabolomics analysis, we identified 1-pyrroline-5-carboxylate (P5C) as a vital molecule that is released by PCC. P5C is responsible for suppressing T cells signaling by increasing ROS and SHP1, and decreasing cytokines and ATP production. We confirmed these findings in vivo, which revealed changed proline dehydrogenase (PRODH) expression in tumor tissues, which in turn influences tumor growth and T cell infiltration.

**Conclusions:**

Our study uncovered a key immunosuppressive axis, which is triggered by PRODH upregulation in PCa tissues, P5C secretion in media and subsequent SHP1-mediated impairment of T cell signaling and infiltration in PCa.

**Electronic supplementary material:**

The online version of this article (10.1186/s40425-018-0466-z) contains supplementary material, which is available to authorized users.

## Background

The original paradigm that tumors are a mass of proliferating cancer cells has now shifted to an in-depth understanding of tumors as complex entities. In addition to cancer cells, tumors harbor a variety of other cell types, including vascular endothelial cells, cancer-associated fibroblasts and various resident or migratory immune cells [1]. The dynamic interaction between cancer cells and immune cells including macrophages, mast cells, neutrophils, T and B cells of the tumor microenvironment critically influences the behavior of tumors [2–4].The importance of T cells in antitumor immunity has been demonstrated in many types of cancer [5, 6]. However, tumors can escape immune attack by various mechanisms of immunosuppression [7, 8].A small number of genes, such as programmed cell death 1 ligand 1 (PD-L1), that enable tumors to evade the immune system have been the focus of intense clinical development efforts [9–11].Reactivating the antitumor responses of T cells by checkpoint blockade has recently been demonstrated to have notable effects on treating cancer, but its response rate needs to be further improved [12, 13].

The past decade has seen a revival of interest to better understand cell metabolism and its association with human tumor. Oncogenes promote, whereas tumor suppressor genes inhibit oncogenesis, however, both influence metabolism [14]. The recent findings in glycolysis, glutaminolysis, serine/glycine metabolism, amino acid metabolism, lipid and membrane lipid metabolism, and TCA cycle enzymes mutations in cancer cells have led to a renewed interest in the field of cancer metabolism [15].

T cells survival and activation also relies on reprogramming of key metabolic pathways and sufficient availability of nutrients like glucose and amino acids [16]. Intratumoral T cells display signs of glucose deprivation and diminished anti-tumor effector functions in glucose-poor tumor microenvironment [17].Similarly intracellular L-arginine concentrations directly impact the metabolic fitness and survival capacity of T cells [18]. Secretion of lactate by tumor cells reduces the activation of T and NK cells, and their production of anti-tumor cytokines such as IFN-γ likely promotes tumor immune evasion and growth [19].

In this study, we focused on the link between the prostate cancer cells (PCC) metabolites with T cell proliferation and functions. We observed that PCC media (PCM) inhibits T cells proliferation and function by increasing the levels of reactive oxygen species (ROS) and decreasing the production of ATP. To uncover the molecular mechanism, we conducted a metabolomics study using liquid chromatography mass spectroscopy (LC/MS-MS) and determined the levels of metabolites in the conditioned media of PCC and normal cells. Our data shows that the levels of1-Pyrroline-5-carboxylate (P5C) was remarkably enhanced in PCM compared to normal cells. Proline dehydrogenase (PRODH) catalyzes the conversion of proline into P5C [20]. PRODH has been identified as one of a few genes that is rapidly and robustly induced by p53. PRODH plays a key role in apoptotic cell death, and autophagy in cancer cells [20]. PRODH is also identified as promising drug target against breast cancer-derived metastasis formation [21]. Importantly, by decreasing the levels of P5C via PRODH knockdown, the proliferation and functions of T cells were recovered. We further uncovered SHP1 as a key regulator of T cell signaling using RNA Seq. These data collectively suggest that decreasing the levels of P5C or inhibiting SHP1 independently or in combination may be a potent way to reactivate T cells signaling for treating prostate cancer (PCa).

## Methods

### Cell preparation and culture

Human primary CD3^+^T cells were isolated from healthy people blood using a CD3εMicroBead kit (Miltenyi Biotec Inc. CA, USA), according to the manufacturer’s instructions. The purity of the CD3^+^ T-cell preparation was assessed by flow cytometry (FACS Calibur, Becton, Dickinson and Company, Franklin Lakes, NJ, USA) using PE-anti-mouse CD3monoclonal antibody (mAb) (eBioscience, Inc., San Diego, CA, USA).

Jurkat cells (human T cell line), HK-2 (human renal tubular epithelial cell line), LNCaP and PC-3 (human prostate cancer cell lines), EL-4 (murine T cell line), and RM-1 (murine prostate cancer cell line), TCMK-1 (murine renal tubular epithelial cell line) were cultured in RPMI1640 (GE Healthcare Life Sciences Hyclone Laboratories, Logan, UT, USA) supplemented with 10% fetal bovine serum (FBS; GE Healthcare Life Sciences Hyclone Laboratores), 10 μg/ml penicillin and 10 μg/ml streptomycin (Beijing Solarbio Science & Technology Co., Ltd. Beijng, China). RWPE-1 cells were cultured in serum free SFM media. The cell culture was maintained in an incubator at 37 °C with 5% CO_2_.

### PCM treatment

Conditioned medium from cultures of PCC cells or non-tumorigenic human renal tubular epithelial and prostate cells was isolated after 48 h. It was centrifuged and the supernatant was harvested.

Primary T cells, Jurkat and EL-4 cells were pretreated with prostate cancer medium (25% final volume in fresh media). T cells were then stimulated with anti-human CD3/CD28 beads in round-bottom 96-well plates.

### Cell proliferation assay

Primary T-cell proliferation was determined by a carboxyfluorescein succinimidyl ester (CFSE) (Life Technologies, Carlsbad, CA, USA) proliferation assay according to the manufacturer’s instructions. In brief, CD3^+^T cells were resuspended in CFSE (5 μM) buffer, incubated at 37 °C with 5% CO_2_ for 20 min, and washed twice in complete medium. The stained T cells, at 2 × 10^5^ cells per well of a 96-well round-bottom plate were then stimulated with anti-human CD3/CD28 beads (2.5 μl/1 × 10^5^ cells) for the indicated times. The cells were then collected and detected with CFSE by flow cytometry (BD FACS Calibur).

Proliferation of the Jurkat and EL-4 cell line was measured using cell counting kit-8 (CCK-8, Dojindo Laboratories, Japan) according to the manufacturer’s protocol.

### Human tissues

All tissue specimens were obtained between June 2016 and July 2017 from 40 patients who underwent surgery for therapeutic treatment at Central Hospital of Wuhan and were without androgen deprivation therapy. There were 25 PC and 15 BPH tissues in all of the specimens. The clinical information on the patients are shown in supplemental table. This study was approved by the ethics committee of Huazhong University of Science and Technology. All patients provided informed consent.

### Flow cytometry

To check the influence of PCM on T cells activation, the expression of CD25 and CD69 was measured by flow cytometry. Samples were stained at 4 °C using CD69-phycoerythrin (PE) or CD25-fluorescein isothiocyanate (FITC) antibodies (BD Biosciences) in FACS buffer (1% BSA in PBS) in the dark for 30 min.The cells were washed twice with cold PBS, resuspended in 200 μl PBS and analyzed using a flow cytometer (BD FACS Calibur). As negative controls, cells were treated with either isotype-matched control antibodies or with no primary antibody. The expression of FOXP3 was detected by FOXP3 Trial Staining Kit, as per manufacturer’s instructions (BD Biosciences).

### Activities of mitochondrial complex I, II and III

The individual activities of mitochondrial complexes I/II/III were measured using the Complex I/II/III Enzyme Activity Kit (GENMED SCIENTIFICS Inc. DE, USA) according to the manufacturer’s instructions.

Complex I activity was measured by following the oxidation of reduced NADH to oxidized NAD^+^, which was assessed by the absorbance at 340 nm. The results were expressed as nmol NADH/min after being normalized to the protein content. Complex II activity was measured by following the conversion of oxidized dichlorophenal-indophenol (DCPIP) to reduced DCPIPH_2_, which was assessed by the absorbance at 600 nm. The results were expressed as nmol DCPIP/min after being normalized to the protein content. Complex III activity was measured by following the conversion of oxidized cytochrome C to reduced cytochrome C, which was assessed by the absorbance at 550 nm. The results were expressed as nmol CoQH2/min after being normalized to the protein content.

### Transfection

Cells were transfected either with PRODH siRNA (100 nM) or non-targeting pool (control siRNA) using lipofectamine 2000 according to the manufacturer’s instructions (Ribo Bio Co., Ltd). After 48 h, the media was collected and then used for the indicated assays. PRODH (designated as siPRODH, target sequences were GATGCAGCGGAAGTTCAAT), and StealthTM RNAi negative controls (designated as siNEG) were purchased from RiboBio.

pcDNA 3.1 expression vectors encoding mouse PRODH were transfected into RM-1 using lipofectamine 2000 reagent according to the manufacturer’s instructions.

The efficiency of the transfection was determined by RT–PCR assay. After 48 h, the media was collected and then used for the indicated assays.

### RNA sequencing

After treatment with PCM for 24 h, CD3^+^T cells were collected to prepare for RNA sequencing. 

Total RNA was extracted and purified using an miRNeasyMini Kit (Qiagen) andchecked for an RNA integrity number to inspect RNA integration by an Agilent Bioanalyzer 2100 (Agilent Technologies) The samples were clustered and sequenced by an Illumina HiSeq 2500 from Mega Genomics Company Limited (Beijing, China).

Prior to analyses, the clean reads were obtained from raw reads which removed low-quality, adaptor-linked, and high content of unknown base (*N* > 1%) reads. And then, the clean reads were mapped on the reference human genome (ftp://ftp.ensembl.org/pub/release-88/fasta/homo_sapiens/dna/Homo_sapiens.GRCh38.dna.toplevel.fa.gz) by using TopHat2. Enrichment of GO (gene ontology) terms was measured. Further, KEGG (Kyoto Encyclopedia of Genes and Genomes) database was used to annotate genes in the metabolic pathway.

Gene expression levels were calculated using Cufflinks version 0.8.0 based on the FPKM. Differential expression was determined using a significance level of FDR (False Discovery Rate) < 0.01under a fold change > 2 or < 0.5.

### Metabolomics analysis

The cultured media of LNCaP, PC-3 and HK-2 cells (1 × 10^5^ per well of a 6 well plate) were harvested after 48 h of culture and stored at − 80 °C. Each group had 10 duplications. Untargeted metabolite profiling was performed using liquid chromatography mass spectroscopy (LC/MS-MS) as previously described [22]. The raw LC-MS data for test set and validation set were loaded in Peak View (ABSciex). Each sample file (including reverse phase and HILIC) was searched against the human metabolome database (HMDB) library loaded in Peak View (ABSciex) and the METLIN database. The robustness of the identification was confirmed by matching the masses of the fragments from the MS-MS spectra for each of the metabolites.

The clean data was obtained by the molecular feature extraction (MFE) tool in the Agilent Masshunter Qualitative Analysis B.04.00 software (Agilent Technologies, USA), then was analyzed by PCA (Principal Component Analysis), PLS-DA (Partial least square-discriminant analysis) and OPLS-DA (Orthogonal Partial least square-discriminant analysis) methods.

Difference between experimental groups was evaluated by unpaired t test (equal or unequal variance) with VIP (Variable Importance in the Projection) > 1 in PLS-DA model. The levels of statistical significant were set at 95% level (*P* < 0.05).

### Animal model

All animal procedures were carried out with the approval of the Animal Ethics Committee of the Huazhong University of Science and Technology. 6-week old male C57BL/6 mice and athymic nude mice were inoculated subcutaneously with 1 × 10^6^ RM-1 cells. After 7 days, 100% of mice grew visible tumors. The two kinds of mice were randomized and assigned to the control, PRODH and PRODH siRNA groups. The tumor volumes were calculated every 3 days using the following equation: tumor volume (mm^3^) = 1/2× (tumor length) × (tumor width)^2^. The weight of the mice was also recorded every 3 days. PRODH cDNA and PRODH siRNA with in vivo-jetPEI Delivery Reagent (Polyplus, France) were intratumorally injected every 3 days for a total of 18 days when the tumor diameter reached 5–7 mm. At the end of experiment, tumors were excised, measured, and then each tumor was fixed in 4% of paraformaldehyde for determining T cells infiltration.

### Statistical analysis

All experiments were performed at least three separate times with data obtained from triplicate wells in each experiment. Data are expressed as means ± SD. The statistical differences between two groups were analyzed by an unpaired Student’s *t-test* (two-tailed); multiple groups were compared using one-way analysis of variance (GraphPad Prism5.0; GraphPad Software; GraphPad, Bethesda, MD). A value of *P* < 0.05 was considered significant.

## Results

### PCC-conditioned media (PCM) inhibits T cell proliferation and impairs cytokine production

**Fig. 1:**
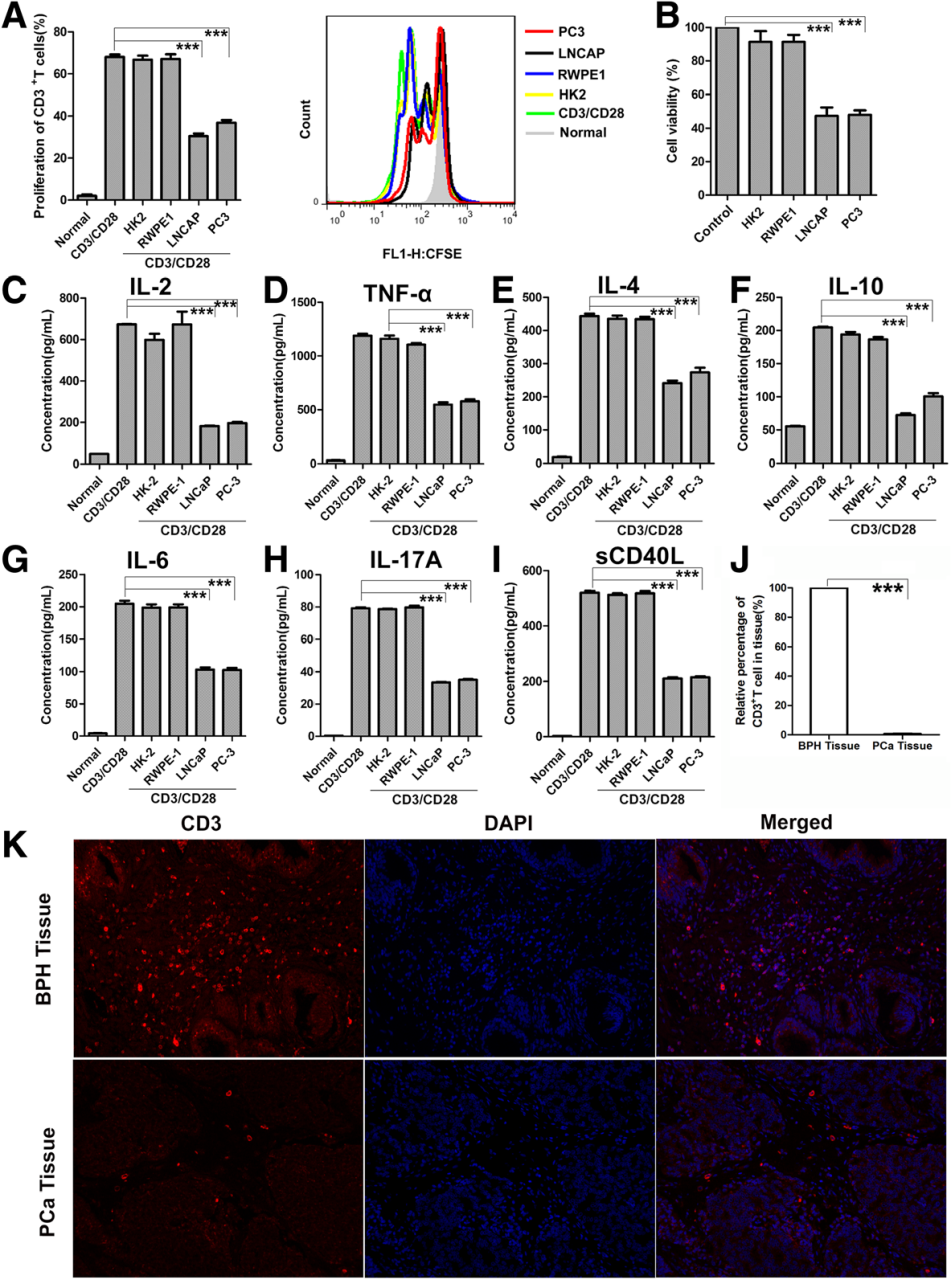
PCM Inhibit T Cell Proliferation, Function and T Cell Infiltration in PC and BPH Tissue. (**a**) CFSE-labeled human primary CD3^+^ T cells were pretreated with PCM or two normal cells media then stimulated for 3 days with anti-CD3/CD28 beads. T-cell proliferation was evaluated by FACS analysis. The right side of bar graph is the representative result of CD3^+^ T cells proliferation. (**b**) Jurkat cells were treated with PCM or two normal cells media for 24 h. Shown is the percentage of cell proliferation by CCK-8 assay. One representative experiment out of three performed. (**c-i**) Human primary CD3^+^ T cells were pretreated with PCM or two normal cells media then stimulated for 3 days with anti-CD3/CD28 beads. Supernatants from cell cultures were analyzed for seven cytokines levels using commercially available ELISA kits. One representative experiment out of three performed. (**j**) Columns showed the quantitative statistics of the infiltration of T cells. (**k**) The infiltration of T cells in PCa (*n* = 25) and BPH (*n* = 15) tissue detected by IF. The red light marked T cells. The representative pictures of IF. Error bars are SEM of biological replicates and ***p < 0.01

To investigate the effect of the metabolites of PCC on T cells, we treated primary human T cells and Jurkat cells with PCM. CD3^+^ T cells were sorted up to > 96% purity from blood of healthy donors (Additional file 1: Figure S1A) and activated using human anti-CD3/CD28 beads. Meanwhile, the T cells were treated with the cultured media of PCC (LNCaP and PC-3) and two normal cells (RWPE1 and HK-2). CFSE labeling cell proliferation assay showed thatCD3^+^ T cells proliferation decreased about 50% in the PCM, whereas the culture media of two normal cells showed little inhibition (Fig. 1a). The same phenomenon was observed in Jurkat cells (Fig. 1b). Meanwhile, we treated Jurkat cells for 6 days to check the duration of PCM, and found that the effect of PCM on Jurkat cells weakened after 3 days (Additional file 1: Figure S1B). Furthermore, when we washed out the PCM and replaced it with fresh media after 24 h, the proliferation of Jurkat cells could be restored (Additional file 1: Figure S1C).

To detect the changes in T cell function in this system, we examined several representative cytokines, which have important roles in tumor and inflammation. To this end, we investigated the levels of IL-2, TNF-α, IL-4, IL-6, IL-10, IL-17a, sCD40L in T cells exposed to either PCM or normal cells media. All of these secreted cytokines were inhibited in T cells exposed to PCM (Fig. 1c-i). To further support the notion that PCC metabolites could affect T cells activation, we measured the expression of CD69 and CD25. However, PCM did not affect the expression of CD69 and CD25 on CD3^+^ T cells, suggesting that T cell activation was not influenced in this system (Additional file 1: Figure S1D, E). We also examined the expression of FOXP3 to check the status of regulatory T cells. As expected, the regulatory T cells also were not affected by PCM (Additional file 1: Figure S1F). Collectively, these data showed that PCM could inhibit the proliferation and function of T cells, but plays no role in T cell activation and regulatory T cells.

We next examined the infiltration of T cells in human PCa tissues and BPH tissues by IF. Our data show that the number of T cells in BPH tissue were significantly higher as compared to PCa tissues, suggesting that T cell infiltration in vivo is inhibited as the cancer progresses (Fig. 1j, k). As our data showed, we postulated that PCa tissues suppress T cells invasion by releasing metabolites in the microenvironment.

### PCC-conditioned medium increases ROS production but decreases ATP production in T cells

ROS and ATP play an importance role in the process of cells growth and proliferation and death. In order to find out whether PCM have effect on ROS and ATP, we inspected their levels in T cells.

**Fig. 2:**
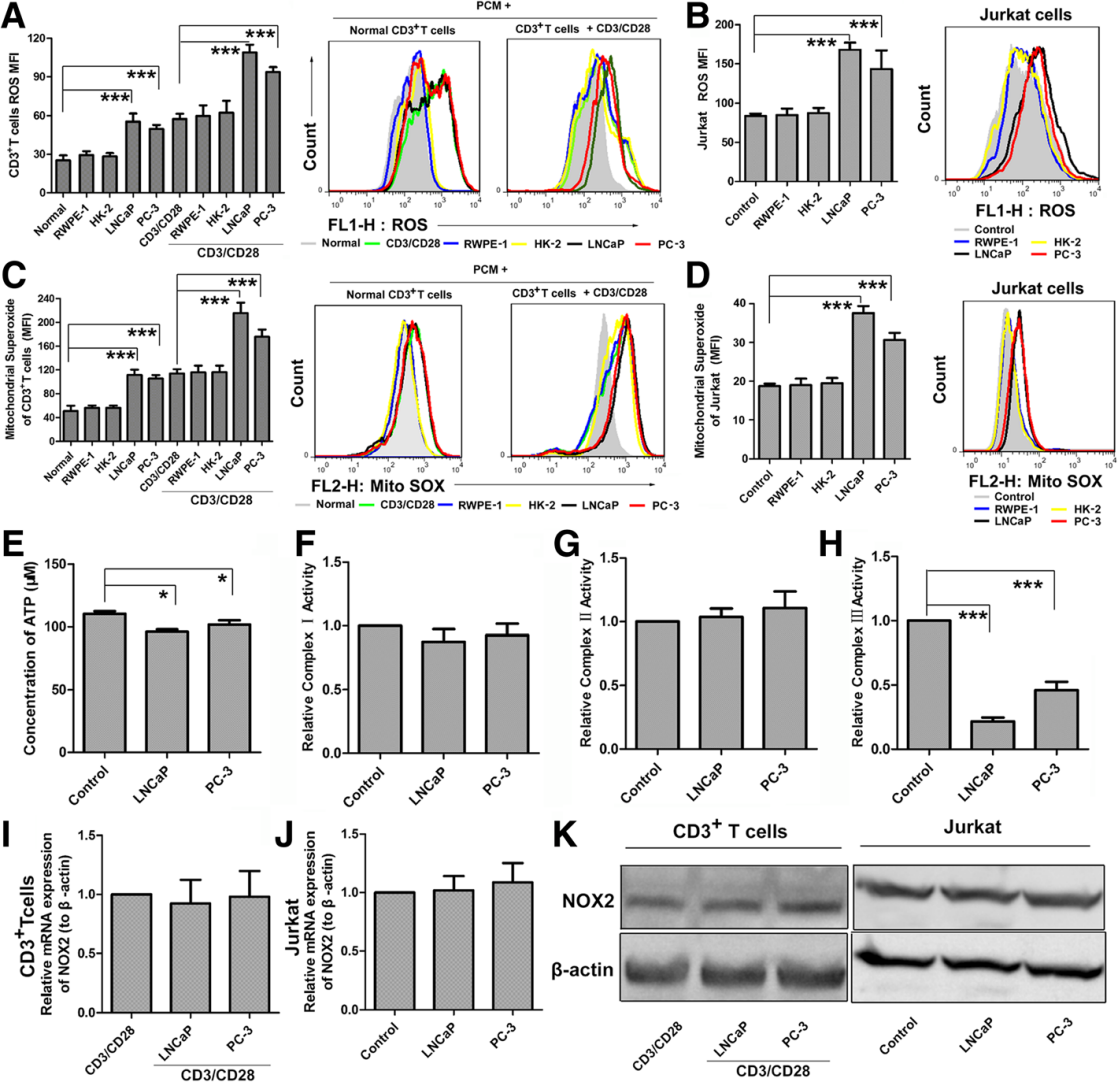
PCM Inhibit T cell ROS and ATP Production and Inhibit Activity of CIII. (**a**) Human primary CD3^+^ T cells were pretreated with PCM or two normal cells media then stimulated for 3 days with or without anti-CD3/CD28 beads. Intracellular total ROS levels were measured by FACS after incubation with the ROS-reactive fluorochorome DCFH. The geometric mean of DCF fluorescence intensity was used to determine the rate of ROS generation. The right side of bar graph is the representative result by flow cytometry. (**b**) Jurkat cells were treated with PCM or two normal cells media for 24 h. Shown is the levels of ROS by flow cytometry. The right side of bar graph is the representative result by flow cytometry. (**c**) Human primary CD3^+^ T cells were pretreated with PCM then stimulated for 3 days with or without anti-CD3/CD28 beads. Shown is the levels of mitochondria ROS by flow cytometry. The right side of bar graph is the representative result by flow cytometry. (**d**) Jurkat cells were treated with PCM or two normal cells media for 24 h. Shown is the levels of mitochondria ROS by flow cytometry. The right side of bar graph is the representative result by flow cytometry. (**e**) Jurkat cells were treated with PCM or two normal cells media for 24 h. Shown is the ATP concentration by microplate reader. (**f**) Jurkat cells were treated with PCM or two normal cells media for 24 h. Shown is the relative activity of CI by microplate reader (**g**) Jurkat cells were treated with PCM or two normal cells media for 24 h. Shown is the relative activity of CII by microplate reader. (**h**) Jurkat cells were treated with PCM or two normal cells media for 24 h. Shown is the relative activity of CIII by microplate reader. (**i, j**) The mRNA expression of NOX2 in CD3^+^ T cells and Jurkat cells after treatment with PCM or two normal cells media by qPCR. (**k**) Western bolt showing the protein expression of NOX2 in CD3^+^ T cells and Jurkat cells. An antibody to β-actin was used as a loading control. All experiments were repeated at least three times. Error bars are SEM of biological replicates and *p < 0.05, ***p < 0.01

We observed that PCM could substantially increase either intracellular total ROS or mitochondrial ROS accumulation both in human CD3^+^ T cells and Jurkat cells (Fig. 2a-d). Importantly, the culture media of two normal cells had no effect on ROS generation. Strikingly, the ATP production in Jurkat cells also could be inhibited by about 15% when cells treated by PCM (Fig. 2e). As high ROS level is toxic to cells, increased ROS and decreased ATP maybe key mechanisms that suppress T cells functions.

### PCC-conditioned medium inhibits complex III of the electron transport chain (ETC)

To discern the main sites of ROS generation in Jurkat cells exposed to PCM, we next analyzed the activities of mitochondria respiratory complexes I, II and III and the expression of catalytic gp91 phox (NOX2). Our data showed that the activities of complexes I (CI) and II (CII) remain unchanged, but the activity of complexes III (CIII) was markedly inhibited by about 70% (Fig. 2f-h). On the other hand, we observed that PCM had no effect on both mRNA and protein expression of NOX2 (Fig. 2i-k). These data suggested that CIII may be the main contributor to ROS production.

To confirm this hypothesis, we initially utilized ROS scavenger N-acetyl cysteine (NAC). With the protection of glutathione precursor NAC, which eliminates intracellular ROS, we observed that the effect of PCM could be weakened. As the levels of ROS were reduced by NAC (Additional file 1: Figure S2A, G), the proliferation of human T cells and Jurkat cells inhibited by PCM were also reversed (Additional file 1: Figure S2D, J). This data suggested that ROS is highly toxic in this system.

We next utilized NOX2 inhibitor apocynin (APO) to find out the effect on the levels of ROS which was increased by PCM and T cells proliferation which was decreased by PCM. As expected, APO had no impact on the levels of ROS (Additional file 1: Figure S2B, H). Accordingly, the inhibition of proliferation of human T cells and Jurkat cells by PCM also remained unchanged (Additional file 1: Figure S2E, K).

To gain insight into the role of mitochondrial respiratory complexes, we investigated the impact of electron transport chain inhibitors on T cells exposed to PCM. Inhibitors of CI and CII, rotenone and thenoyltrifluoroacetone (TTFA), respectively, did not show any effect on ROS levels or cell viability. However, the inhibitor of CIII, antimycin A, enhanced the effect of PCM notably on ROS levels (Additional file 1: Figure S2C, I) and T cells proliferation (Additional file 1: Figure S2F, L). Collectively, these data confirmed that the inactivation of CIII is crucial for increase in ROS production, which is highly toxic to T cells. We also analyzed T cell function in the presence of NAC and antimycin A by ELISA. Strikingly, NAC reverses the influence of PCM on T cells cytokines secretion which was further enhanced by antimycin A (Additional file 1: Figure S3).

### RNA-Seq revealed PCM triggers SHP1 upregulation in T cells

**Fig. 3:**
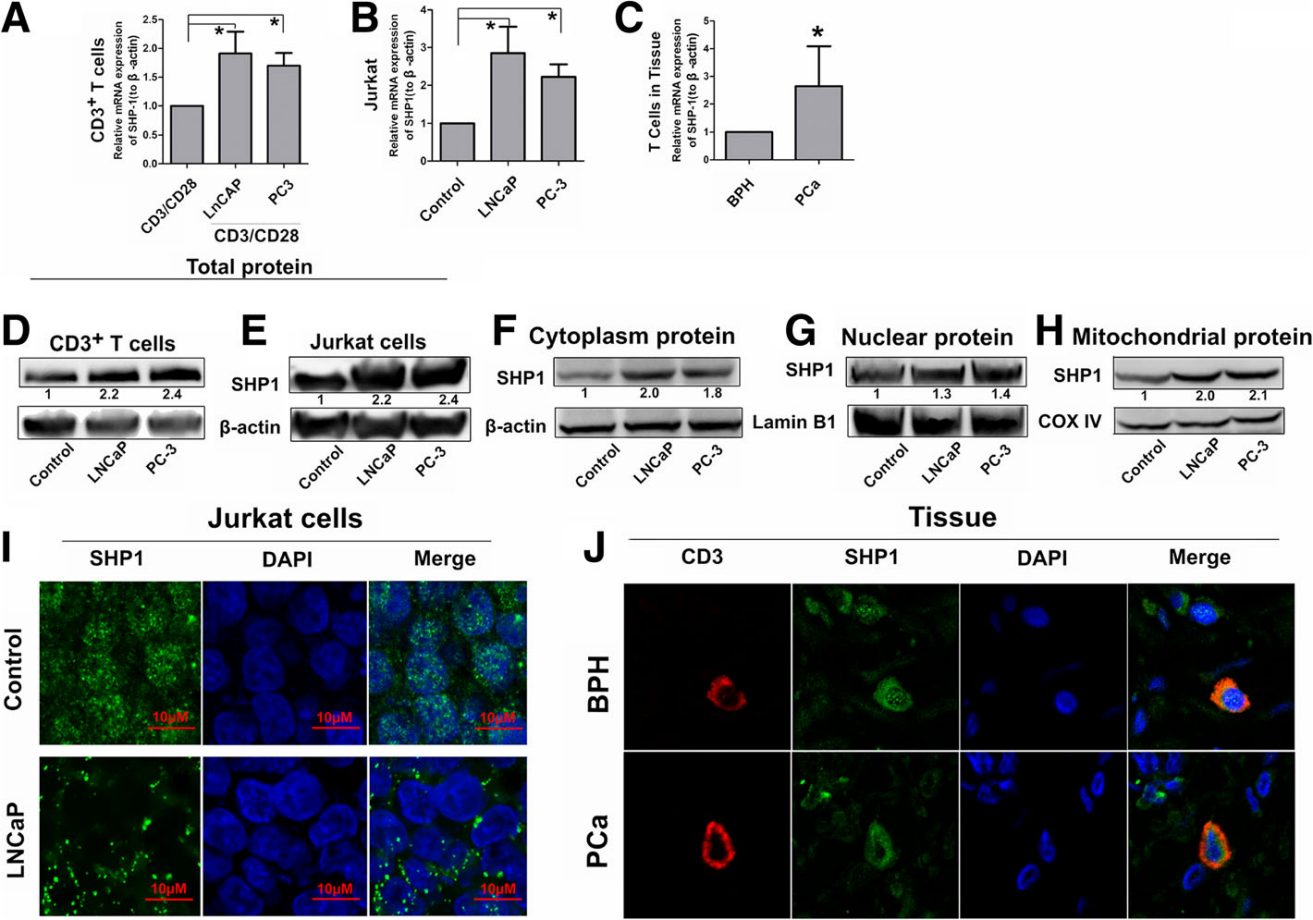
The Effect of PCM on Expression and Translocation of SHP1 in T cells. (**a-b**) The mRNA expression of SHP1 in CD3^+^ T cells and Jurkat cells after treatment with PCM by qPCR. (**c**) Obtained T cells from human prostate tissue by laser capture microdissection, and checked SHP1 expression in T cells of BPH and PCa tissue by qPCR. (**d-e**) Western bolt showing the protein expression of SHP1 in CD3^+^ T cells and Jurkat cells after treatment with PCM. An antibody to β-actin was used as a loading control. (**f**) Western bolt showing the cytoplasm protein expression of SHP1 in Jurkat cells after treatment with PCM. An antibody to β-actin was used as a loading control. (**g**) Western bolt showing the nuclear protein expression of SHP1 in Jurkat cells after treatment with PCM. An antibody to Lamin B1 was used as a loading control. (**h**) Western bolt showing the mitochondria protein expression of SHP1 in Jurkat cells after treatment with PCM. An antibody to COX IV was used as a loading control. (**i**) The localization of SHP-1 in Jurkat cells after treatment with PCM by immunofluorescence. DAPI was used for marking nuclear. (**j**) The localization of SHP-1 in T cells of PCa and BPH tissue by confocal microscopy. DAPI was used for marking nuclear, and CD3 for marking T cells Error bars are SEM of biological replicates and *p < 0.05

To elucidate the mechanism by which PCM inhibit T cell survival, we first examined T cells total mRNA levels by RNAseq. Our data showed that following treatment with PCM, the levels of 1564 genes changes, including downregulation of 709 genes and upregulation of 855 genes (Additional file 1: Figure S4B). Then we analyzed the expression of all the genes involved in the TCR signaling (Additional file 1: Figure S4C). We focused on SHP1, a negative regulator of TCR-mediated signaling in T cells, which was upregulated in T cells exposed to PCM. We verified this phenomenon by qPCR and western blotting. After treatment with PCM, we observed that both mRNA (Fig. 3a-b) and protein (Fig. 3d-e) levels of SHP1 were upregulated in human T cells and Jurkat cells which accorded with RNAseq result.

### SHP1 levels are increased in T cells which are infiltrated in PCa clinical specimens compared to T cells in BPH clinical tissues

Furthermore, using laser capture microdissection, we acquired the T cells in human prostate tissues. Compared with BPH tissue, the mRNA levels of SHP1 in T cells in PCa tissue were significantly higher (Fig. 3c). Previous studies have shown that endogenous SHP1 in Jurkat cells and primary T cells is cytoplasmic both before and after TCR stimulation [23]. In order to identify the expression of SHP1 in different subcellular locations, we first detected the levels of SHP1 in untreated control cells, which showed cytoplasmic, mitochondrial and nuclear localization. However, PCM treatment remarkably increased the levels of SHP1 in the cytoplasm and the mitochondria while SHP1levels in the nucleus increased to a smaller extent (Fig. 3f-h).

Next, we analyzed the subcellular localization of SHP1 by confocal microscopy, which revealed that intranuclear localization of SHP1 in Jurkat cells is decreased significantly by PCM (Fig. 3i). We then verified this phenomenon inhuman tissue specimens. Compared to BPH tissue, the SHP1 intranuclear localization in T cells of PCa tissues was much lower (Fig. 3j). Together, these results indicate that SHP1 is upregulated by PCM mainly in the cytoplasm and the mitochondria.

### SHP1 inhibition restores complex III activity, which weakens the effect of PCC metabolites on T cells

**Fig. 4:**
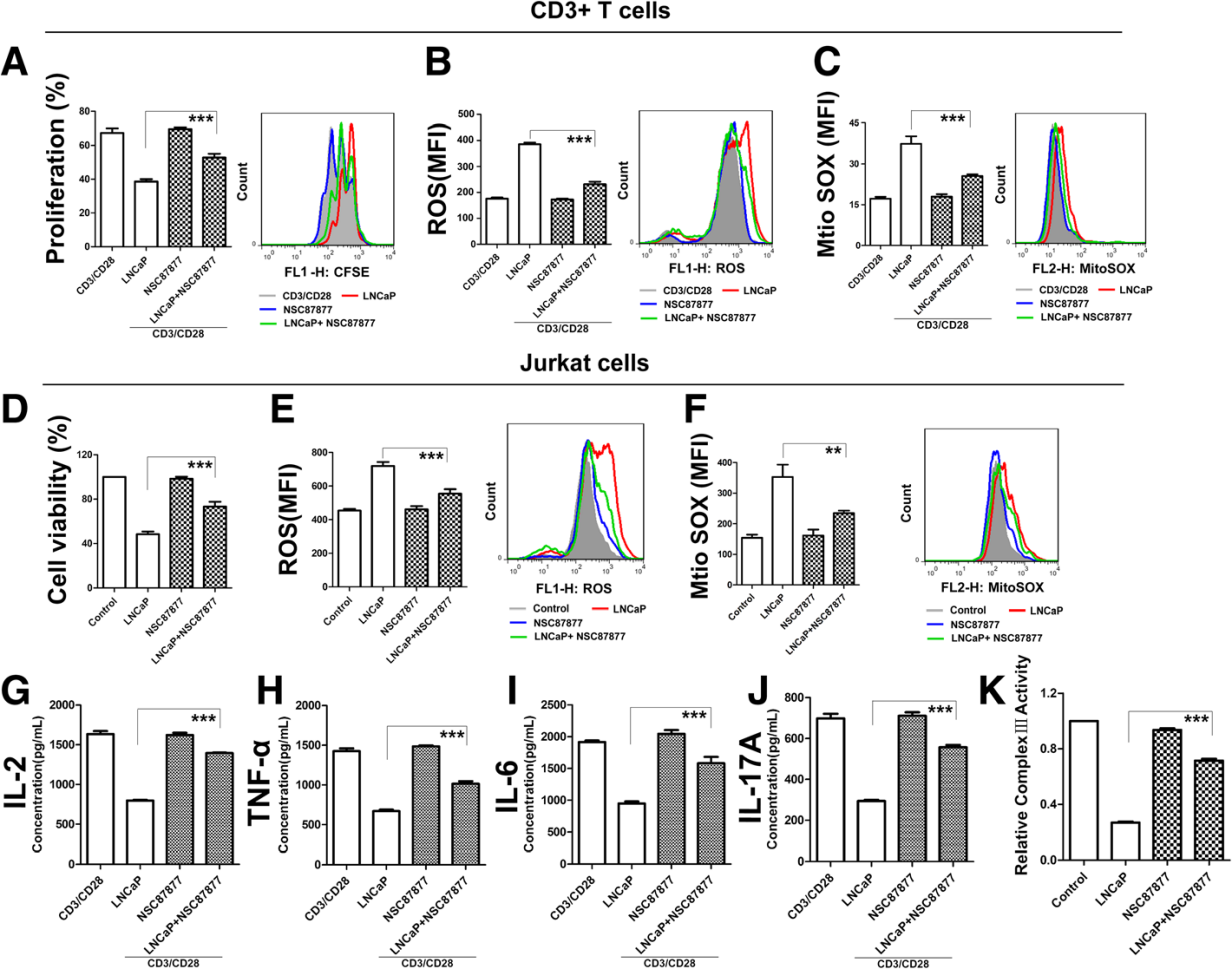
SHP1 Inhibition Weaken the Effect of PCC Metabolites on T Cells. (**a**) Human primary CD3^+^ T cells were pretreated with PCM and the inhibitor of SHP1 NSC87877 (12.5 μM) then stimulated for 3 days with anti-CD3/CD28 beads. Shown is the percentage of cell proliferation by flow cytometry. The right side of bar graph is the representative result by flow cytometry. (**b, c**) Human primary CD3^+^ T cells were pretreated with PCM and the inhibitor of SHP1 NSC87877 (12.5 μM) then stimulated for 3 days with anti-CD3/CD28 beads. Shown is the levels of total and mitochondria ROS by flow cytometry. The right side of bar graph is the representative result by flow cytometry. (**d**) Jurkat cells treated with PCM and the inhibitor of SHP1 NSC87877 for 24 h. Shown is the percentage of cell proliferation by CCK-8 assay. (**e, f**) Jurkat cells treated with PCM and the inhibitor of SHP1 NSC87877 for 24 h. Shown is the percentage of cell proliferation by CCK-8 assay. The right side of bar graph is the representative result by flow cytometry. (**g-j**) Human primary CD3^+^ T cells were pretreated with PCM and the inhibitor of SHP1 NSC87877 (12.5 μM) then stimulated for 3 days with anti-CD3/CD28 beads.. Supernatants from cell cultures were analyzed for cytokines levels using commercially available ELISA kits, including IL-2, TNF-α, IL-6, IL-17A. (**k**) Jurkat cells treated with PCM and the inhibitor of SHP1 NSC87877 for 24 h. Shown is the relative activity of CIII by microplate reader. All experiments were repeated at least three times. Error bars are SEM of biological replicates and **p < 0.01; ***p < 0.001

To address whether SHP1 plays a key role in T cell inhibition, we utilized NSC87877, an inhibitor of SHP1. Using a dose-dependent study, we initially determined the optimal concentration of NSC87877, which does not inhibit T cells proliferation. Conditioned media which contained SHP1 inhibiter, restored T cell proliferation to a large extent, and reduced ROS in human T cells and Jurkat cells compared to control conditioned media (Fig. 4a-f). Likewise, the function of T cells secreted cytokines were also restored due to SHP1inhibition (Fig. 4g-j). In order to analyze the relationship of SHP1 and CIII, we also detected the activity of CIII in NSC87877-treated PCM. Through the inhibition of SHP1, the inactivation of CIII induced by PCM could be reversed (Fig. 4k). Collectively, these data demonstrate that SHP1 is vital for the process of PCM-induced suppression of T cells.

We next utilized wild type (WT) and SHP1 knockout (SHP^+/−^) C57 mice, and isolated CD3+ T cells from spleens. We treated T cells with the media of murine prostate cancer cells (RM-1). Importantly, the proliferation of WT T cells was inhibited and the production of ROS was increased, but the media of RM-1 have little effect on SHP^+/−^T cells (Additional file 1: Figure S5), thereby underscoring a central role of SHP1 in T cell suppression.

### LC-MS/MS revealed high levels of 1-Pyrroline-5-carboxylate (P5C) in PCC conditioned media compared to normal cells

**Fig. 5:**
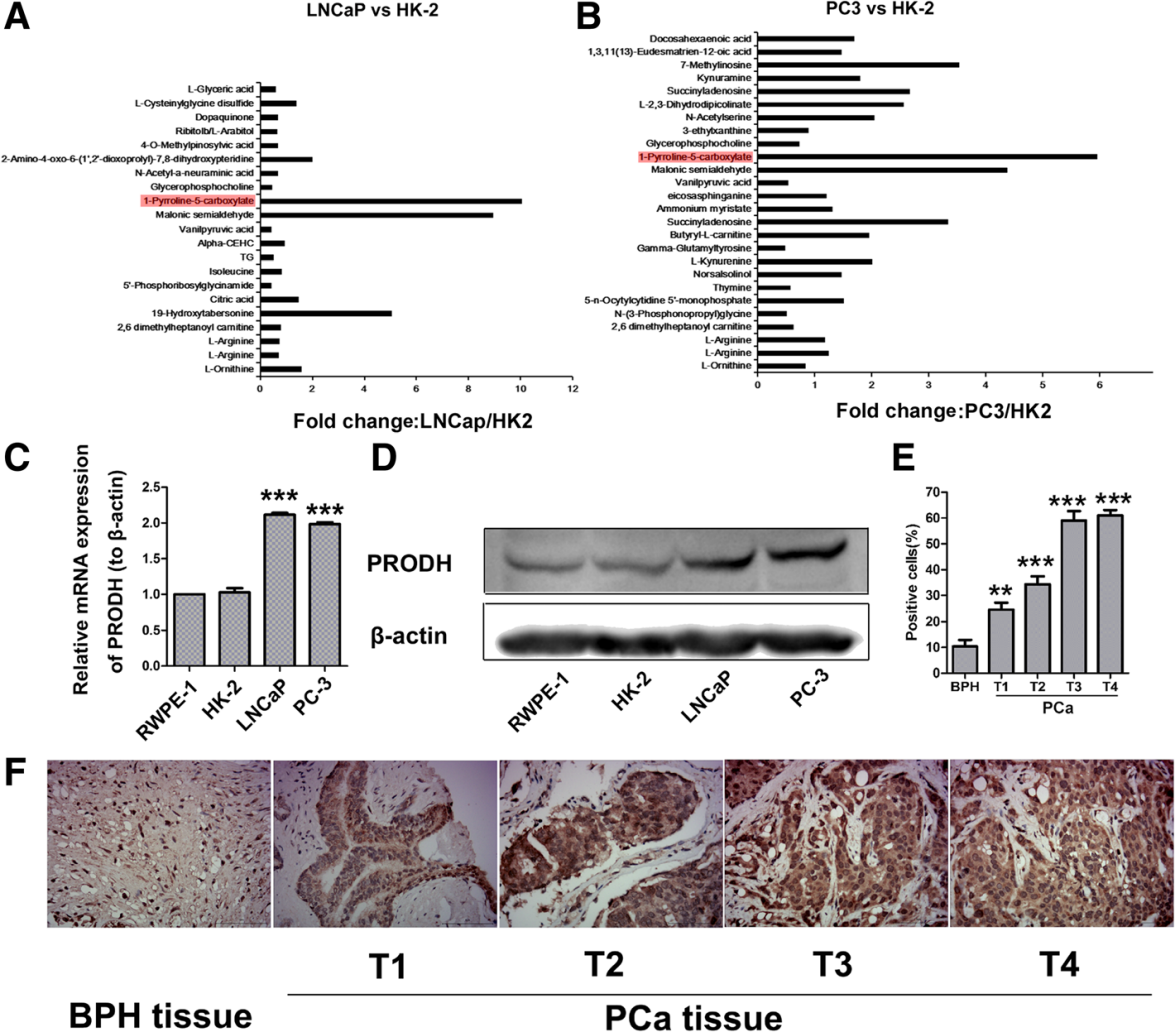
Difference in Metabolites and PRODH Expression Between PCC and Normal Cell. (**a**) All identified and analyzed different metabolites between LNCaP and HK-2 cell. The content of P5C (red marked) in LNCaP cultured media obviously exceeded the content in HK-2. (**b**) All identified and analyzed different metabolites between PC3 and HK-2 cell. The content of P5C (red marked) in PC3 cultured media obviously exceeded the content in HK-2. (**c-d**) The mRNA and protein expression of PRODH in four cell lines. (**e**) The expression of PRODH in PCa and BPH tissue. Columns are expressed as mean ± SD. ** *P* < 0.05; ***P < 0.01. (**f**) The expression of PRODH in PCa and BPH tissues by immunohistochemical

To elucidate the mechanism by which PCC metabolites suppress T cell survival and upregulates SHP1, we first examined the diversity of metabolites between PCC and normal cell through LC-MS/MS. Because of the media of RWPE1 is different with PCC cells, which may result in false positives, we only analyzed the metabolites in LNCaP, PC-3 and HK-2 cells. As shown in Fig. 5a, we first compared the differences between LNCaP and HK-2, and identified 21 metabolites, which exhibited different levels. Especially, the content of P5C (red marked) in LNCaP culture media exceeded the content in HK-2. Similarly, there were 26 variant molecules between PC-3 and HK-2, and the most remarkable molecule was again P5C (Fig. 5b). Therefore, we speculated that P5C may be the most important metabolite, which causes T cells inhibition and dysfunction.

### PRODH is overexpressed in PCa clinical specimens and correlates with disease progression

Proline dehydrogenase (PRODH) converts proline into P5C [20]. In the subsequent step, P5C dehydrogenase (P5CDH) converts P5C into glutamate. We thus investigated the expression of PRODH indifferent cell lines, and found that both the mRNA and protein levels of PRODH were significantly higher in LNCaP and PC-3 compared to RWPE1 and HK-2 cells (Fig. 5c, d).

In order to further validate our inferences, we also examined the expression of PRODH in human prostate cancer (PCa) and benign prostatic hyperplasia (BPH) tissues. 25 tumor tissues samples and corresponding 15 BPH tissues samples were analyzed by IHC. Compared with corresponding non-neoplastic tissue, the expression of PRODH was up-regulated in all of the tumor tissues (Fig. 5f). Then we compared the expression of PRODH in different stages of PCa. We found that the PRODH expression were significantly higher in the advanced tumors (Fig. 5e). But there was no statistical significance between T4 and T3, even when the expression in T4 was higher. Collectively, these data provided us a hypothesis that P5C, as a metabolite of PCC, may be a key hazardous substance for T cells.

### PRODH knockdown of PCC weakens the effect on T cells

**Fig. 6:**
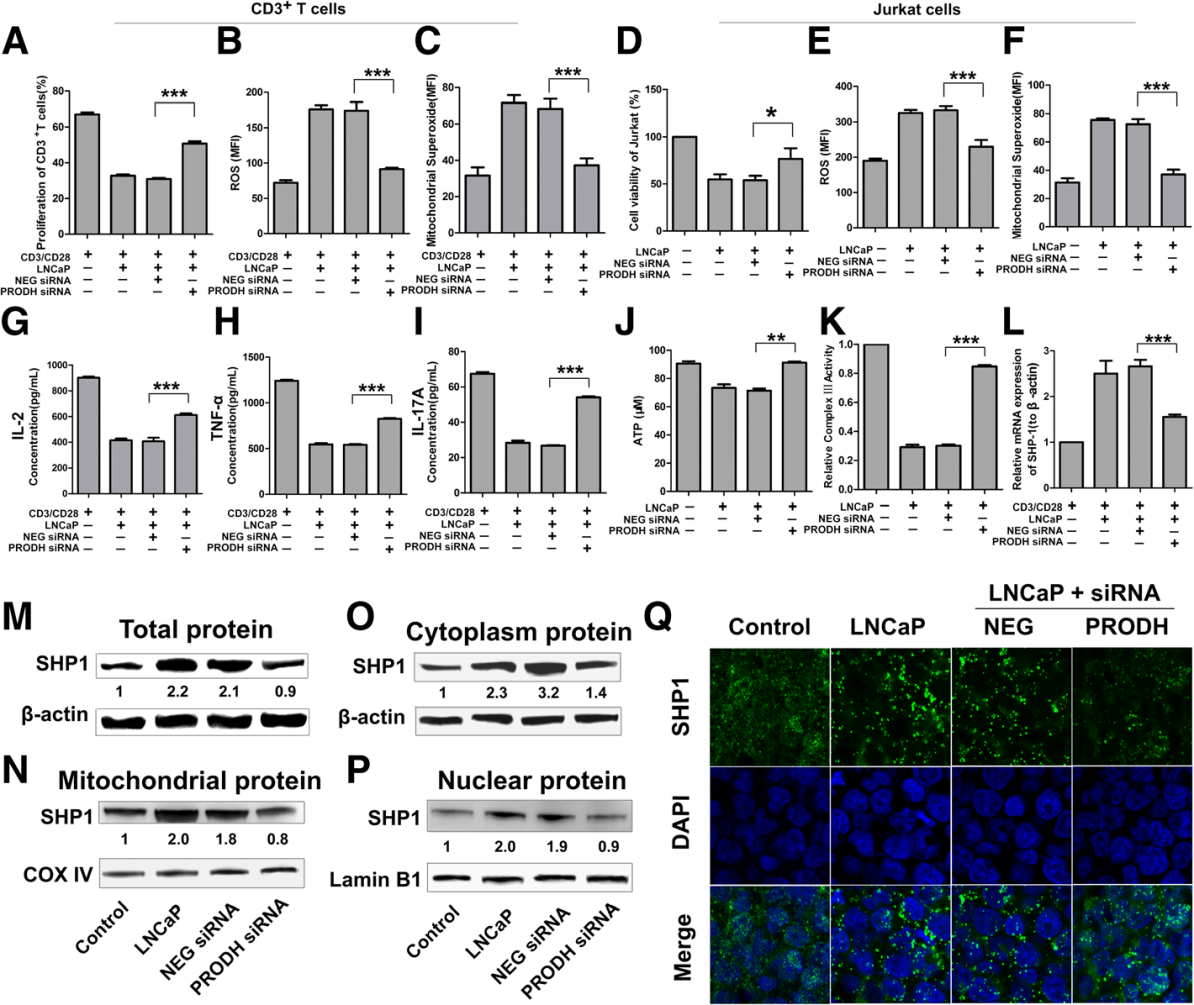
PRODH Knockdown of PCC Weaken the Effect on T Cells. (**a**) Human primary CD3^+^ T cells were pretreated with PCM or PRODH knockdown-PCM then stimulated for 3 days with anti-CD3/CD28 beads. Shown is the percentage of cell proliferation by flow cytometry. (**b**) Human primary CD3^+^ T cells were pretreated with PCM or PRODH knockdown-PCM then stimulated for 3 days with anti-CD3/CD28 beads.. Shown is the levels of ROS by flow cytometry. (**c**) Human primary CD3^+^ T cells were pretreated with PCM or PRODH knockdown-PCM then stimulated for 3 days with anti-CD3/CD28 beads. Shown is the levels of mitochondria ROS by flow cytometry. (**d**) Jurkat cells treated with PCM or PRODH knockdown-PCM for 24 h. Shown is the percentage of cell proliferation by CCK-8 assay. (**e-f**) Jurkat cells treated with PCM or PRODH knockdown-PCM for 24 h. Shown is the levels of total and mitochondria ROS by flow cytometry.(**g-i**) Human primary CD3^+^ T cells were pretreated with PCM or PRODH knockdown-PCM then stimulated for 3 days with anti-CD3/CD28 beads. Supernatants from cell cultures were analyzed for cytokines levels using commercially available ELISA kits, including IL-2, TNF-α, IL-17A. (**j-k**) Jurkat cells treated with PCM or PRODH knockdown-PCM for 24 h. Shown is the ATP concentration and activity of CIII (**l**) The mRNA expression of SHP1 in Jurkat cells by qPCR after treatment with PCM or PRODH knockdown-PCM. (**m**) Western bolt showing the protein expression of SHP1 in Jurkat cells after treatment with PCM or PRODH knockdown-PCM. (**n-p**) Western bolt showing the cytoplasm, mitochondria and nuclear protein expression of SHP1 in Jurkat cells after treatment with PCM or PRODH knockdown-PCM. (**q**) The localization of SHP-1 in Jurkat cells after treatment with PCM or PRODH knockdown-PCM by immunofluorescence. DAPI was used for marking nuclear. All experiments were repeated at least three times. Error bars are SEM of biological replicates and *p < 0.05; **p < 0.01; ***p < 0.001

To elucidate the effect of P5C on T cells, we knocked down PRODH using siRNA to decrease the content of P5C in PCM. With the transfection of PRODH siRNA, the PRODH expression in LNCaP cells was down-regulated significantly (Additional file 1: Figure S7A). We then examined the proliferation of LNCaP by CCK-8 assay, and found that cell growth was not affected by PRODH knockdown, which excluded the decrease of P5C in PCM caused by cell number (Additional file 1: Figure S7B). Moreover, we also checked the production of P5C in PRODH knockdown cell lines, and we found that PRODH knockdown decreased the production of P5C (Additional file 1: Figure S7C). Subsequently, we used the PCM after PRODH knockdown to treat T cells, and discovered that the effect of PCM on T cells could be weakened. With PRODH knockdown, the influence on the inhibition of cell growth and the production of ROS in both human CD3^+^T cells and Jurkat cells were weaker than the PCM with non-targeting siRNA (Fig. 6a-f). Similarly, the function of T cells in secreting cytokines were also recovered by PRODH knockdown in PCC (Fig. 6g-i). Similarly, the effect on the inhibition of ATP generation (Fig. 6j) and CIII activity (Fig. 6k) showed the same trend. We also evaluated SHP1expression and translocation in T cells treated with PRODH siRNA. We found that the expression of SHP1 in T cells treated with PRODH knockdown was much lower. Further, PRODH knockdown inhibited the translocation of SHP1 to the mitochondria and the cytoplasm (Fig. 6m-p). Next, we also verified that SHP1 intranuclear localization was enhanced upon PRODH knockdown, compared to non-targeting siRNA PCM (Fig. 6q). Collectively, these data verified the hypothesis that P5C elimination could weaken the effect of PCM on T cells.

In order to show P5C is sufficient to cause the aforementioned phenotype, we directly added P5C into the media of CD3^+^ T cells. Because the solvent of P5C should be HCL, we used HCL as the control. We found that P5C could inhibit the proliferation by about 25%, and also could increase the generation of ROS (Additional file 1: Figure S7D, E).

### The change of PRODH expression effect the growth of tumor and T cells infiltration on animal model

**Fig. 7:**
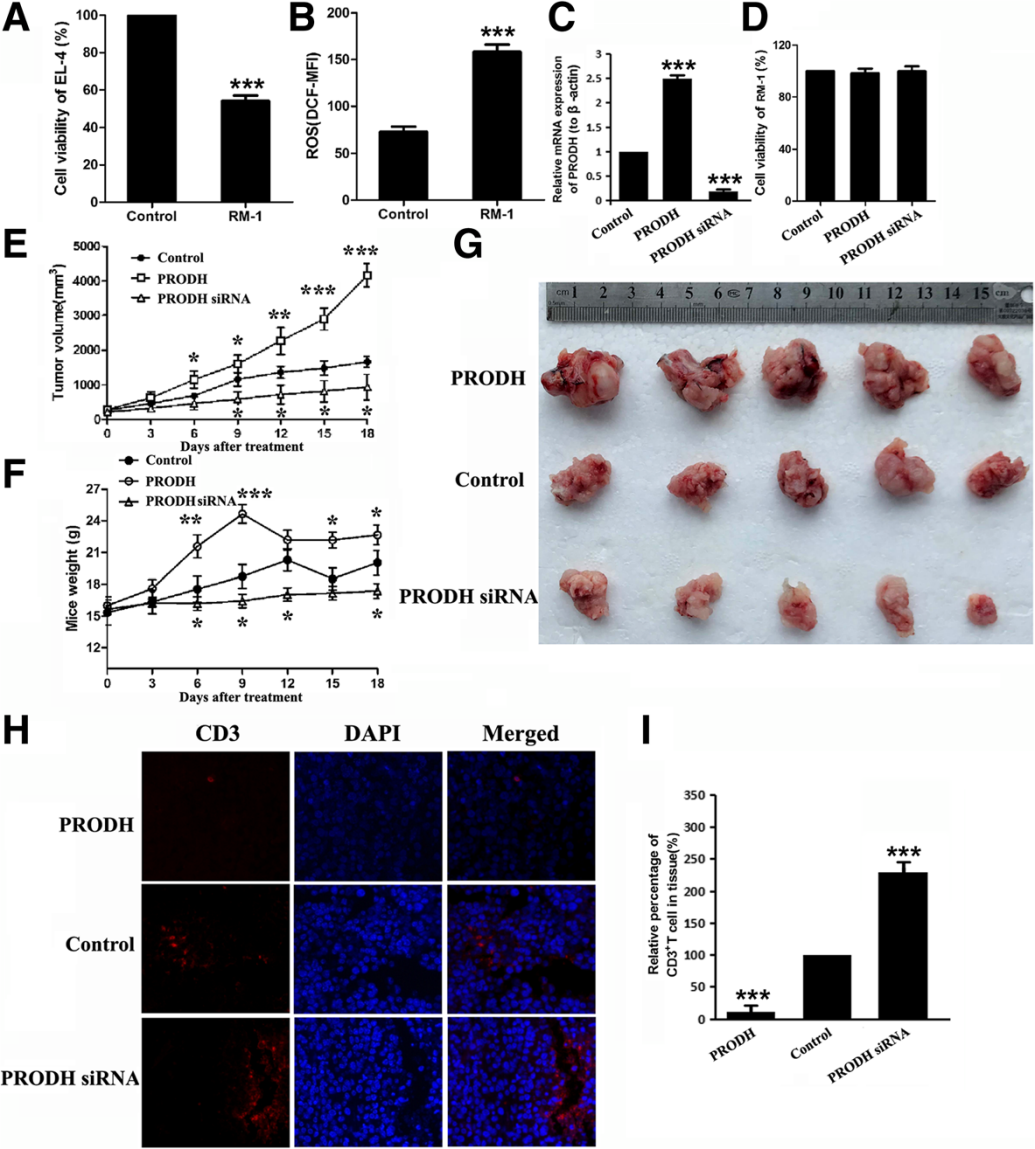
The Change of PRODH Expression Affect the Growth of Tumor and T cells Infiltration in vivo. (**a**) EL-4 cells were treated with RM-1 cultured media for 24 h. Shown is the percentage of cell proliferation by CCK-8 assay. One representative experiment out of three performed. (**b**) EL-4 cells were treated with RM-1 cultured media for 24 h. Shown is the levels of ROS by flow cytometry. (**c**) The expression of PRODH in RM-1 after transfection by qPCR. (**d**) The cell number of RM-1 after transfection by CCK-8 assay. (**e**) Mean of tumor volume measured at the indicated number of days after mice were treated. (**f**) Mean of body weight of mice measured at the indicated number of days after mice were treated. (**g**) The picture of tumors after harvesting. **(h)** The infiltration of CD3^+^ T cells in tumor tissue detected by IF. The red light marked T cells. (**i**) Columns showed the quantitative statistics of the infiltration of T cells. Error bars are SEM of biological replicates and *p < 0.05; **p < 0.01; ***p < 0.001

Because the defectiveness of T cells in nude mice, we could not use human prostate cancer cells to construct xenograft model to verify our findings in vivo. So we chose murine prostate cancer cells RM-1 to construct animal model. Primarily, we treated murine T cells EL-4 with RM-1 cultured media, and we observed the same phenomenon that the proliferation of EL-4 was inhibited by about 50% and the production of ROS increased by two times (Fig. 7a, b). We also upregulated and downregulated PRODH in RM-1 cells, and found that the change of PRODH expression did not affect cancer cells growth (Fig. 7d). In the meantime, we detected that the content of P5C in RM-1 was higher than the murine normal cells (TCMK-1). With the changes of PRODH expression in RM-1, the content of P5C in RM-1 media also altered (Additional file 1: Figure S8A).

Strikingly, the changes in PRODH expression influenced tumor growth in animal model (Fig. 7g). The upregulation of PRODH increased tumor growth, and PRODH knockdown reversed it. Furthermore, the changes in PRODH expression also impacted CD3^+^ T cells infiltration in tumors (Fig. 7h, i). The upregulation of PRODH decreased T cells infiltration, and PRODH knockdown accelerated T cells infiltration. Not only in CD3^+^ T cells, we also observed the same phenomenon in CD4^+^ and CD8^+^ T cells (Additional file 1: Figure S8 B-E).

We also constructed xenograft model in nude mice, where the change in PRODH expression in RM-1 have no effect on tumor growth (Additional file 1: Figure S8 F-H), suggesting that the effect is mediated through T cells.

## Discussion

It is well accepted that tumor microenvironment impair immune cell functions both directly and indirectly [24]. In order to directly suppress immunity through regulatory ligands, tumor cells create a microenvironment that is metabolically hostile to effector lymphocytes [25]. Tumors deplete nutrients and accumulate waste products, such as lactate or kynurenine that directly inhibit T cells [17, 19]. Nevertheless, how the metabolites of cancer cells released in the extracellular milieu affect T cells is still incompletely understood.

Our study shows that the metabolites of PCC inhibits the proliferation and function of T cells by increased ROS accumulation and decreased ATP generation. ROS play significant role as important innate effector by controlling infection and tumorigenesis as well as by modulating T-cell reactivity and autoimmunity [26]. The current consensus is that low levels of ROS are beneficial, facilitating adaptation to stress via signaling, whereas high levels of ROS are deleterious because they trigger oxidative stress [27]. Thus, we hypothesized that ROS may be the culprit that triggers the dysfunction of T cells when exposed to metabolites of PCC. ROS can be generated by both enzymatic and nonenzymatic systems, including mitochondria and NADPH oxidases (NOX) complexes, in the intracellular as well as in the extracellular space [28, 29]. The NOX family of enzymes consists of seven members (NOX 1–5 and two dual oxidases, DUOX 1 and 2). NOX2 is an important source of ROS in T cells as NOX2-deficient T cells display strongly reduced ROS production [30]. In mitochondria, the main sites of ROS generation are respiratory complexes I (aka NADH: ubiquinone oxidoreductase, CI), II (aka succinatecoenzyme Q reductase, CII) and III (aka ubiquinol: cytochromec oxidoreductase, CIII) [31]. Our findings showed that NOX2, CI and CII have no role in ROS generation, while CIII inactivity increases ROS production. Blocking CIII could generate conditions for reverse electron transfer (RET) which is associated with the generation of high levels of ROS [27]. Therefore, CIII is the main source of ROS production in T cells in PCC microenvironment.

In order to elucidate the mechanism by which PCM inhibits T cell survival, we detected human T cells total mRNA levels by RNAseq. A striking finding is that after treatment of the metabolites of PCC the levels of 1564 genes changed, out of which 709 genes were down-regulated and 855 genes were up-regulated. Some key genes involved in TCR signaling were also altered, such as PD-1, CTLA-4, SHP1, LCK and ZAP70. As the negative regulator of TCR-mediated signaling in T cells, the expression of PD-1 and SHP1 was upregulated, but CTLA-4 showed opposite result. SHP1 is a negative regulator, acting, at least in part, directly or indirectly through the inactivation of src-family kinases [32]. c-Src is also present in the mitochondria and its inhibition directly inhibits mitochondrial electron transport and promotes ROS production with pathological consequences [33]. Furthermore, in addition to inactivating Src, it is possible that SHP1 might also dephosphorylate respiratory chain phosphotyrosines after transfer to the mitochondrial innermembrane [34]. Thus, we hypothesized that SHP1 may be responsible for the high levels of ROS. Interestingly, we observed that the mRNA and protein levels of SHP1 in T cells are up-regulated after the treatment of the metabolites of PCC. Meanwhile, the nuclear localization of SHP1 in T cells is decreased by PCM, but it is increased in the mitochondria and cytoplasm. In this study, to determine whether SHP1 is required for CIII inhibition and ROS generation, which are induced by the metabolites of PCC, we used SHP1 inhibitor NSC87877. SHP1 inhibition reverses the effect of PCM. Therefore, our finding supports the hypothesis that SHP1 plays a key role in the dysfunction of T cells when they are exposed to metabolites of PCC.

Using mass spectrometry, we further analyzed the differences in the metabolites between PCC and normal cells. Because of the media of RWPE1 cell line is different compared to PCC, which may result in false positives, we chose PC-3, LNCaP and HK-2 cells. Interestingly, the levels of P5C in the metabolites of PC-3 and LNCaP was remarkably high compared to HK-2 cells. P5C, an N-substituted imino acid, is an intermediate not only in proline biosynthesis but also in its catabolism [35]. PRODH catalyzes the conversion of proline to P5C, which is then converted to glutamate by pyrroline-5-carboxylate dehydrogenase (P5CDH) in mitochondria. It was reported that accumulation of P5C is responsible for mitochondrial ROS production and hence cell death in yeast [36]. Hence, we assumed the P5C may be the key hazardous substance for T cells. Thus, we decreased the content of P5C by down-regulating the expression levels of PRODH. PRODH, as a mitochondrial inner membrane enzyme is involved in the first step of proline catabolism and has been identified as double-edged sword, which functions either as tumor suppressor to initiate ROS-mediated apoptosis, or as tumor survival factor through ATP production or ROS-induced autophagy depending on the tumor microenvironment [20, 37–39]. It was reported that targeting PRODH activity could have the potential to be effective against cancer cells and micrometastases [21]. PRODH are identified as a direct transcriptional targets of p53 [40], which is a tumor suppressor and express low activity in cancer. We found that the expression of PRODH was increased in PCa tissues compared to human BPH tissues. Similar results were obtained in normal and PC cell lines. Thus, we speculate that PRODH may also be regulated by other factors, which will be explored in future.

In our study, PRODH knockdown did not inhibit the proliferation of PCC, but it reversed the harmful effect of P5C on T cells, including proliferation, function and ROS production. Meanwhile, the expression and translocation of SHP1 in T cells was also restored. We also verified our findings in animal model, where PRODH over-expression enhanced tumor growth and decreased T cells infiltration in tumors, and PRODH knockdown showed opposite phenomenon.

## Conclusion

In conclusion, P5C released into tumor environment by PCC inhibits the proliferation and function of T cells, by up-regulating SHP1. SHP1 inhibits CIII and promotes the generation of ROS. The harmful effect of P5C on T cell survival and anti-tumor functionality may be exploited therapeutically to improve adaptive T cell therapies. Additionally, our study on the interplay between tumor metabolism and T cells of the tumor microenvironment provides a new perspective for immunosuppression and a new standpoint for tumor immunotherapy.

## Additional file

Additional file 1: **Figure S1.** The Effect of PCM on T Cells and Jurkat cells, Related to Fig. 1. **Figure S2.** ROS Scavenger and Inhibitor of CIII Could Weaken the Effect of PCM on T cells. **Figure S3.** ROS Scavenger and Inhibitor of CIII Could Weaken the Effect of PCM on T cells. **Figure S4.** The Media of RM-1 Have No Effect on SHP1 Knockdown T Cells. **Figure S5.** Quality Control of the RNAseq, Rlated to Fig. 3. **Figure S6.** Quality Control of the Metabonomics, Rlated to Fig. 5. **Figure S7.** The Efficiency of PRODH Knockdown and the Effect to Cell Number, and the Effect of Additional P5C on Human CD3^+^ T Cells, Related to Fig. 6. **Figure S8.** The Change of PRODH Expression Affect CD4^+^ and CD8^+^ T cells Infiltration in vivo Which Have no Influence on Nude Mice Xenograft. **Table S1.** The clinical information on the patients. (DOCX 3226 kb)
