# Supplementary material for: 1-Pyrroline-5-carboxylate released by prostate Cancer cell inhibit T cell proliferation and function by targeting SHP1/cytochrome c oxidoreductase/ROS Axis
Source: J Immunother Cancer. 2018 Dec 13;6:148. doi: 10.1186/s40425-018-0466-z (PMC6291986; doi:10.1186/s40425-018-0466-z)
Supplement: Supplementary file 1 — Figure S1. The Effect of PCM on T Cells and Jurkat cells, Related to Fig. 1. Figure S2. ROS Scavenger and Inhibitor of CIII Could Weaken the Effect of PCM on T cells. Figure S3. ROS Scavenger and Inhibitor of CIII Could Weaken the Effect of PCM on T cells. Figure S4. The Media of RM-1 Have No Effect on SHP1 Knockdown T Cells. Figure S5. Quality Control of the RNAseq, Rlated to Fig. 3. Figure S6. Quality Control of the Metabonomics, Rlated to Fig. 5. Figure S7. The Efficiency of PRODH Knockdown and the Effect to Cell Number, and the Effect of Additional P5C on Human CD3+ T Cells, Related to Fig. 6. Figure S8. The Change of PRODH Expression Affect CD4+ and CD8+ T cells Infiltration in vivo Which Have no Influence on Nude Mice Xenograft. Table S1. The clinical information on the patients. (DOCX 3226 kb) [file 40425_2018_466_MOESM1_ESM.docx]

## **Supplementary Methods**

**Mice**

Six-week-old male C57BL/6 mice and athymic nude mice (BALB/c-nu/nu mice) were obtained from Chinese Academy of Sciences Laboratory Animal Center (Laboratory Animal, Beijing, China). Six-week-old male SHP1 knockout mice (SHP1^+/-^) were kindly provided by Prof. Li Zeng. Mice were maintained in specific pathogen-free conditions.

**Cell proliferation measurements**

T-cell proliferation was determined by a carboxyfluorescein succinimidyl ester (CFSE) (Life Technologies, Carlsbad, CA, USA) proliferation assay conducted according to the manufacturer's instructions. In brief, CD3^+^ T cells were resuspended in CFSE (5µM) buffer, incubated at 37°C with 5% CO_2_ for 20 min, and washed twice in complete medium. The stained T cells, at 2×10^5^ cells per well of a 96-well round-bottom plate, were then stimulated to proliferate with anti-CD3/CD28 beads for the indicated times. The cells were then collected and detected for CFSE by flow cytometry (BD FACSCalibur).

Proliferation of the Jurkat and EL-4 cell line was measured using cell counting kit-8 (CCK-8, Dojindo Laboratories, Japan) according to the manufacturer's protocol.

**Cytokine analysis**

T cells on 96-well plates were incubated with or without conditioned media followed by stimulation with CD3/CD28 beads for 72h. Supernatants from cell cultures were analyzed for cytokine levels using commercially available ELISA kits (Human IL-2, TNFα, IL-4, IL-6, IL-10, IL-17A, IFNγ and sCD40L), according to the manufacturer’s instructions (eBioscience Inc. CA, USA).

**Measurement of cellular ROS**

Intracellular ROS levels of T cells or Jurkat or EL-4 cells were measured using DCFH-DA probes (10μM) according to the manufacturer’s instructions (Reactive Oxygen Species Assay kit, Beyotime Biotechnology, Shanghai, China). In brief, cells were harvested and incubated with 10 μM of DCFH-DA in RPMI 1640 (without supplements) for 20 min at 37 °C. They were then washed by centrifugation at 300g for 5 min, and fluorescence was detected using flow cytometry (BD, FACSCalibur). The geometric mean (Gmean) of the DCF fluorescence intensity was used to determine the rate of ROS generation.

**Mitochondrial ROS detection**

Mitochondrial ROS level was measured useing MitoSOX Red. Cells were incubated with 200µl MitoSOX (5 mM/L) for 15 min at 37°C. After washing, cells were collected and detected by flow cytometry. The geometric mean (Gmean) of the fluorescence intensity was used to determine the rate of Mitochondrial ROS generation.

**ATP detection**

After treatment, Jurkat cells were homogenized with the lysis buffer and centrifuged at 12000 ×g for 10 minutes. According to the manufacturer’s instructions, ATP levels were measured by ATP Colorimetric/Fluorometric assay kit (BioVison, Inc, CA, USA). The OD value of the standards and the samples were captured at 570 nm using a microplate reader. Meanwhile, the amount of ATP was calculated based on the standard curve.

**Mitochondrial isolation**

After treatments, Jurkat cells were collected and washed twice via centrifugation with ice cold PBS and then cell pellets were lysed using the Mitochondria Isolation Kit according to the manufacturer’s instructions (GENMED SCIENTIFICS Inc. DE, USA). Total mitochondrial protein concentrations were quantified using the BCA kits and kept at－80°C.

[**Immunofluorescence**](file:///D:\%E5%B7%A5%E5%85%B7\%E5%BA%94%E7%94%A8%E8%BD%AF%E4%BB%B6\%E6%9C%89%E9%81%93\Dict\7.5.2.0\resultui\dict\?keyword=immunofluorescence)

After treatment, Jurkat cells were fixed with 4 % paraformaldehyde (Fisher; Fair Lawn, New Jersey) for 20 min at room temperature, and blocked with normal goat serum. Primary antibodies were used at 1:200 dilutions and HRP-conjugated anti-rabbit or anti-mouse (1:1000 dilution) were used as secondary antibodies. Specific binding of antibodies was detected by FITC or PE. The subsequent counterstained for nuclei was 4′, 6-diamidino-2-phenylindole (DAPI).

Formalin-fixed and paraffin-embedded tissue sections (5µm) were dewaxed with xylene and rehydrated through an ethanol gradient into water. Following blocking of endogenous peroxidase activity with 0.3% hydrogen peroxide for 10 min, the sections were washed with PBS and incubated over-night with primary antibodies. Primary antibodies were used at 1:200 dilutions and HRP-conjugated anti-rabbit or anti-mouse (1:1000 dilution) were used as secondary antibodies. Specific binding of antibodies was detected by FITC or PE. The subsequent counterstained for nuclei was DAPI.

All the Images were acquired with an Olympus BX61 ﬂuorescence microscope system.

### Laser capture microdissection and mRNA extraction

### The fresh BPH and PCa tissues were embedded over liquid nitrogen and held at −80°C. After 24 h, tissues from the molded blocks were sliced onto the polyethylene naphthalate (PEN) membrane slides that allow for the Arcturus™ Laser Capture Microdissection (LCM) system. Samples were sliced into 8 μm sections according to manufacturer’s instructions (Thermo Scientific, IL, USA). All procedures were performed under RNase-free conditions. Subsequently, the microscope laser cut the tissue and catapulted the T cells into the hovering adhesive cap. From each used slide 30–150 cells were collected. After microdissection, the adhesive caps were sterilely removed from the plastic and placed into 200μl lysis buffer. The cap and lysis buffer were incubated at 42°C for 30 min and then stored at −80°C until RNA extraction. Total RNA was extracted, amplified, and underwent realtime-RNA using PicoPure RNA Isolation Kit protocols.

**Real-time RT-PCR analysis**

Total RNA was harvested using an RNeasy Mini Kit (Qiagen). RNA (500 ng) was reverse transcribed into cDNA using a High-Capacity cDNA Reverse Transcription Kit (Life Technologies). The cDNA was synthesized using the SuperScript II Reverse Transcriptase Kit (Invitrogen). Taqman primers for NOX2, SHP1 and PRODH were purchased from Applied AuGCT. Ampliﬁcation was performed at 95°C for 10min, followed by 40 cycles of 15 s at 95°C and 1min at 60°C. Relative gene expression levels were calculated using 2^(-ΔΔCt)^ methods.

**Western blot analysis**

Total protein was prepared in RIPA lysis buffer with the addition of mammalian protease inhibitor cocktail (Sigma) and phosphatase inhibitor. Cytoplasm and nuclear protein were isolated using Nuclear Protein Extraction Kit following the manufacture’s instruction. Mitochondrial protein obtained from mitochondria isolation. The protein concentrations were measured by BCA protein assay. Equal amounts of cell lysates were separated using SDS-polyacrylamide gels and transferred by electroblotting onto a Polyvinylidene fluoride (PVDF) membrane. The following primary antibodies were used: rabbit anti-NOX2, SHP1 and PRODH. Membranes were then incubated with the horseradish peroxidase-conjugated secondary antibodies (1:4000; Abcam). One hour later, the membranes were washed three times with TBST, and then visualized using DAB detection system. The mouse anti-β-actin, Lamin B1 and COXⅣ were used as total and cytoplasm, nuclear and mitochondrial protein normalization respectively.

**Immunohistochemistry (IHC)**

Formalin-fixed and paraffin-embedded tissue sections (5 µm) were dewaxed with xylene and rehydrated through an ethanol gradient into water. Following blocking of endogenous peroxidase activity with 0.3% hydrogen peroxide for 10 min, the sections were washed with phosphate buffered saline (PBS) and incubated over-night with rabbit PRODH antibody at the dilution of 1:200 in a humidified chamber at 4°C. After washing with PBS, sections were incubated with biotinylated secondary antibody for 30 min at 37°C and then with horseradish peroxidase labeled streptavidin for 30 min at 37°C. Diaminobenzidine (DAB) was used as chromogen and the sections were subsequently counterstained with hematoxylin, then dehydrated, cleared and mounted.

**P5C assay with o-AB**.

To 1.35 ml of sample and 1.5 ml of 10% trichloroacetic acid were added 0.15 ml of 0.1 M o-AB in 40% (v/v) ethanol. After 25 min at room temperature, the sample was cleared by centrifugation (1×10^4^g, 10min), and the absorbance in a l-cm cuvette at 440 nm was determined. The molar extinction coefficient of P5C was 2580[1].

**References**

1. Mezel V. A., Knox, W. E.: **Properties and analysis of a stable derivative of pyrroline-5-carboxylic acid for use in metabolic studies**. Anal. Biochem. 1976, 74:430–440.

**Supplementary Figures**


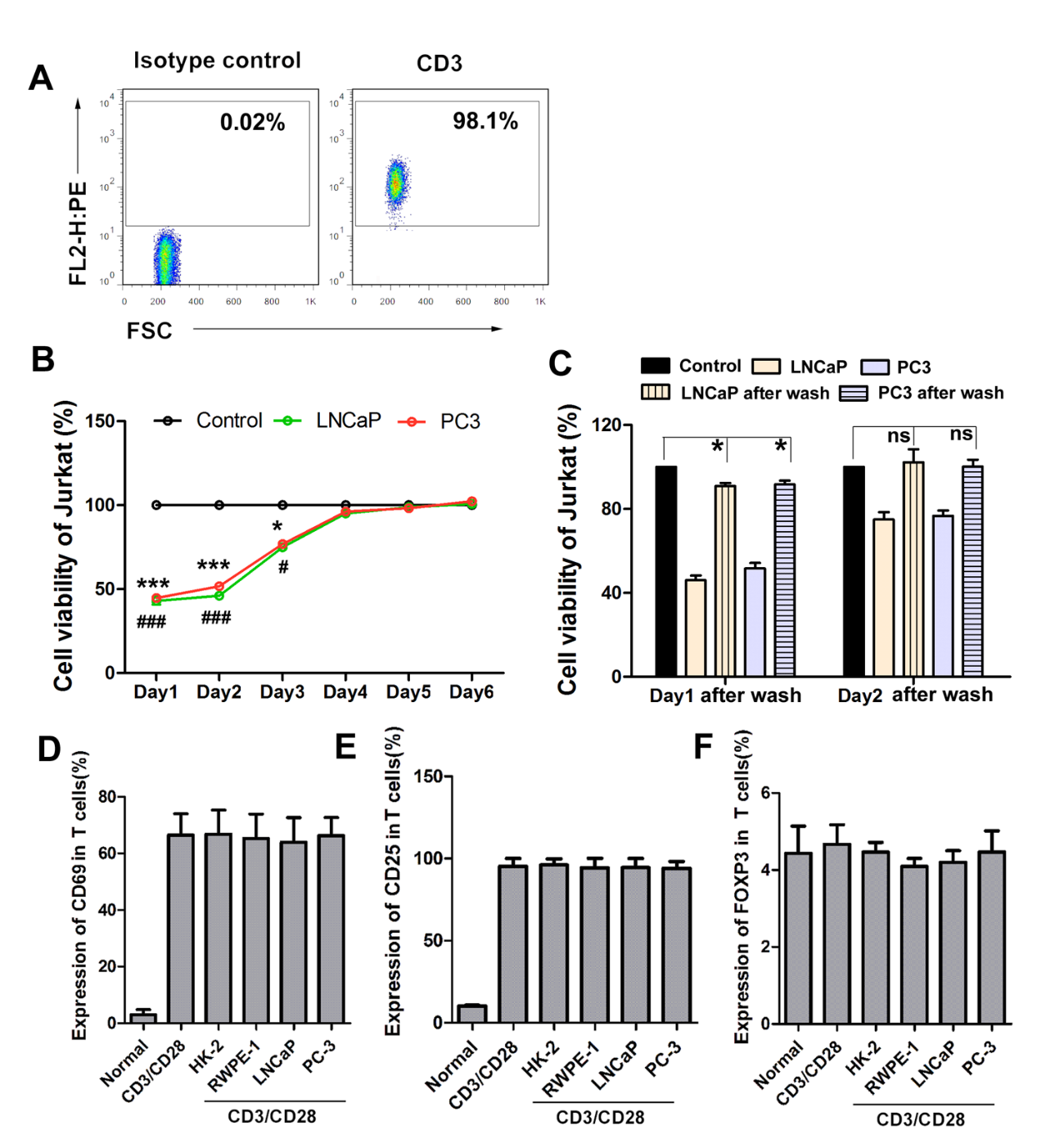


**Figure S1. The Effect of PCM on T Cells and Jurkat cells, Related to Figure 1**

(A) Sorting of human naive CD3^+^ T cells. Shown are FACS plots of cells after enrichment with magnetic beads.

(B) Jurkat cells were treated with PCM for 6 days to check the duration of PCM.

(C) Washed out the PCM and replaced on fresh media to check whether the proliferation of Jurkat cells could be restored.

(D, E) Human CD3^+^ T cells were activated for 72h and treated with PCM or two normal cells media. Shown is the expression of CD69 and CD25 by flow cytometry.

(F) Human CD3^+^ T cells were activated for 72h and treated with PCM or two normal cells media. Shown is the expression of FOXP3 by flow cytometry.

All experiments were repeated at least three times.

The mean ± SEM is reported (**P<*0.05; ns, *P*>0.05; n=3 per group).





**Figure S2. ROS Scavenger and Inhibitor of CIII Could Weaken the Effect of PCM on T cells**

(A) Human CD3^+^ T cells were activated for 72h and treated with PCM and ROS scavenger NAC (500 nM). Shown is the levels of ROS detected by flow cytometry.

(B) Human CD3^+^ T cells were activated for 72h and treated with PCM and the inhibitor of NOX2 Apocynin (5 μM). Shown is the levels of ROS detected by flow cytometry.

(C) Human CD3^+^ T cells were activated for 72h and treated with PCM and the inhibitor of CI (1 nM), CII (1 nM) and CIII (2 nM). Shown is the levels of ROS detected by flow cytometry.

(D) Human CD3^+^ T cells were activated for 72h and treated with PCM and ROS scavenger NAC. Shown is the percentage of cell proliferation detected by flow cytometry.

(E) Human CD3^+^ T cells were activated for 72h and treated with PCM and the inhibitor of NOX2 Apocynin. Shown is the percentage of cell proliferation detected by flow cytometry.

(F) Human CD3^+^ T cells were activated for 72h and treated with PCM and the inhibitor of CI, CII and CIII. Shown is the percentage of cell proliferation detected by flow cytometry.

(G ) Jurkat cells treated with PCM and ROS scavenger NAC for 24h. Shown is the levels of ROS detected by flow cytometry.

(H) Jurkat cells treated with PCM and Apocynin for 24h. Shown is the levels of ROS detected by flow cytometry.

(I) Jurkat cells treated with PCM and the inhibitor of CI, CII and CIII for 24h. Shown is the levels of ROS detected by flow cytometry.

(J) Jurkat cells treated with PCM and ROS scavenger NAC for 24h. Shown is the percentage of cell proliferation detected by CCK-8 assay.

(K) Jurkat cells treated with PCM and Apocynin for 24h. Shown is the percentage of cell proliferation detected by CCK-8 assay.

(L) Jurkat cells treated with PCM and the inhibitor of CI, CII and CIII for 24h. Shown is the percentage of cell proliferation detected by CCK-8 assay.

All experiments were repeated at least three times. Error bars are SEM of biological replicates and ***p* < 0.01, ****p* <0.001.





**Figure S3. ROS Scavenger and Inhibitor of CIII Could Weaken the Effect of PCM on T cells**

(A-D) Human CD3^+^ T cells were activated for 72h and treated with PCM and ROX scavenger. Supernatants from cell cultures were analyzed for cytokines levels using commercially available ELISA kits, including IL-2, IL-6, IL-17A, TNF-α.

(E-H) Human CD3^+^ T cells were activated for 72h and treated with PCM and the inhibitor of CIII. Supernatants from cell cultures were analyzed for cytokines levels using commercially available ELISA kits, including IL-2, IL-6, IL-17A, TNF-α.

All experiments were repeated at least three times. Error bars are SEM of biological replicates and **p* < 0.05; ** *P* <0.01; ****P<*0.001.


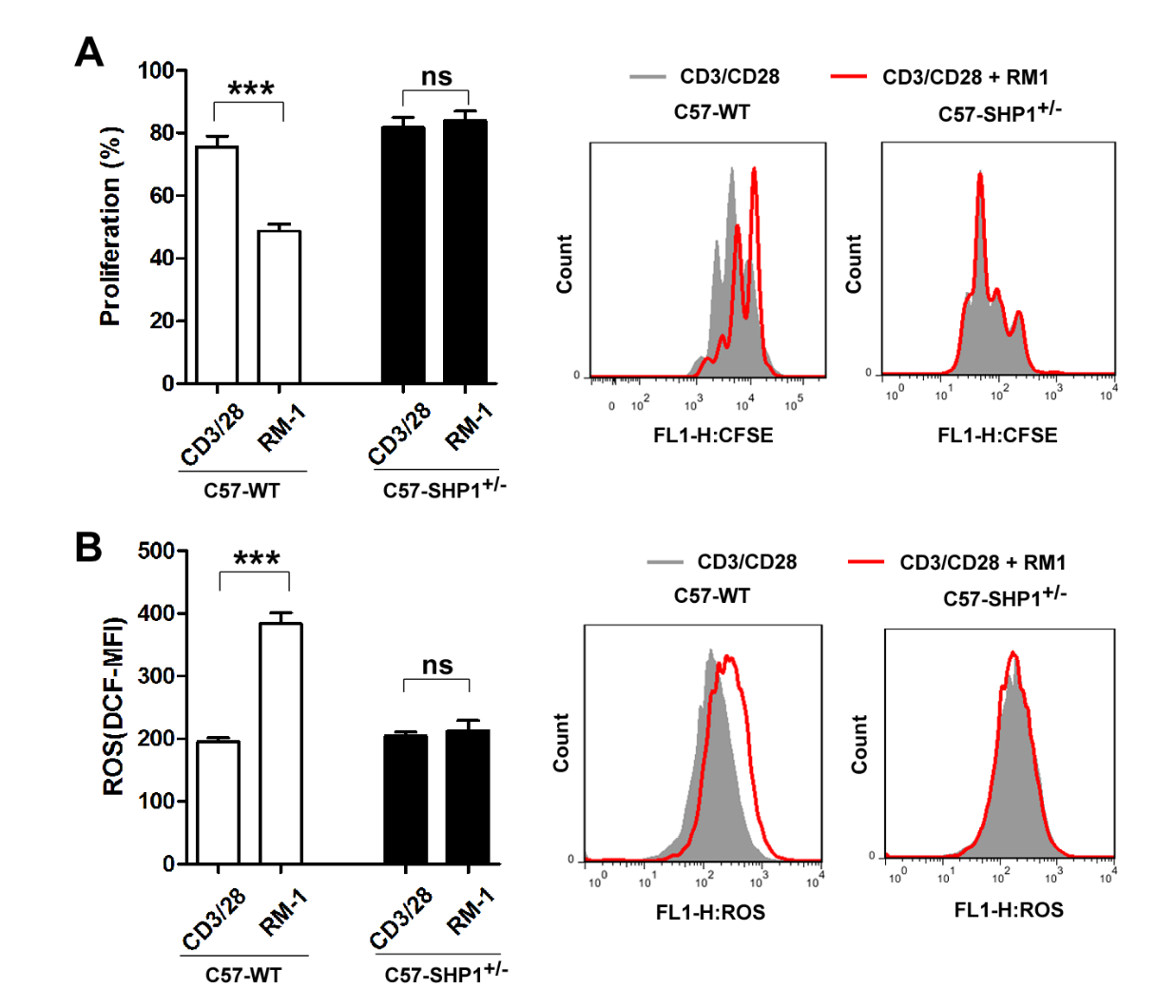


**Figure S4. The Media of RM-1 Have No Effect on SHP1 Knockdown T Cells**

(A) WT and SHP1^+/-^ CD3^+^ T cells were activated for 72h and treated with the media of RM-1. Shown is the percentage of cell proliferation by flow cytometry. One representative experiment out of three performed. The right side of bar graph is the representative result of CD3^+^ T cells proliferation examined by flow cytometry.

(B) WT and SHP1^+/-^ CD3^+^ T cells were activated for 72h and treated with the media of RM-1. Shown is the levels of ROS by flow cytometry. The right side of bar graph is the representative result by flow cytometry.

Error bars are SEM of biological replicates and ****P<*0.001; ns, *P*>0.05


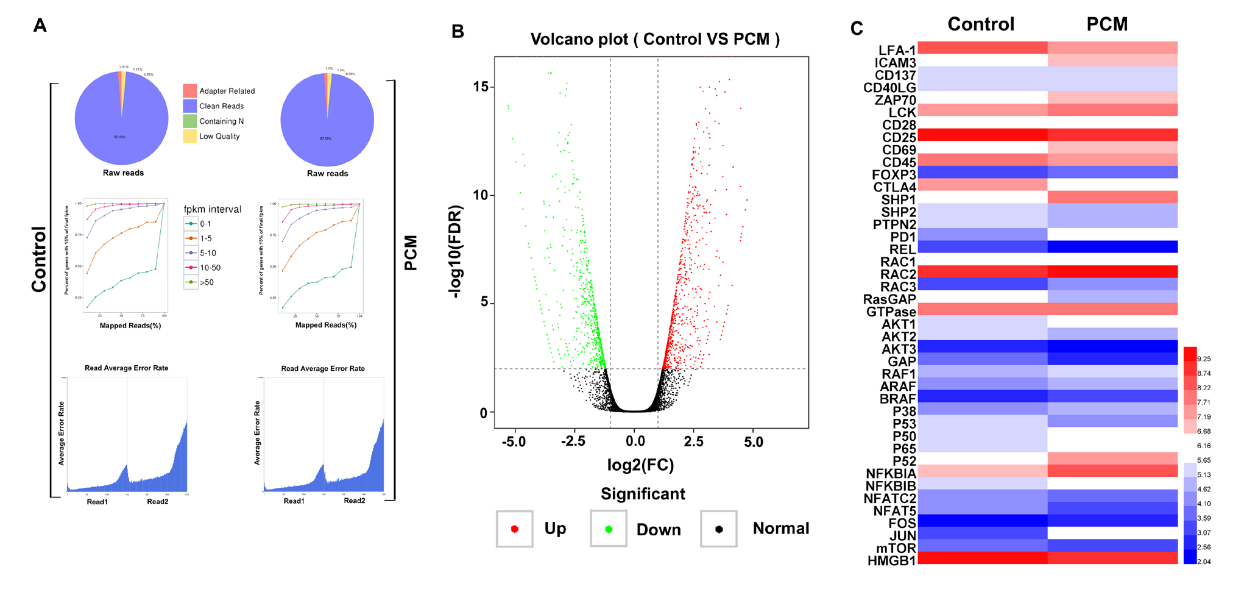


**Figure S5. Quality Control of the RNAseq, Rlated to Figure 3**

(A) Show the raw reads, mapped reads and read avarage error rate.

(B) The volcano plot to show the date of RNAseq.

(C) Human CD3^+^ T cells were activated for 72h and treated with PCM. The heat map shows the relative expression of some genes involved in the TCR signaling.


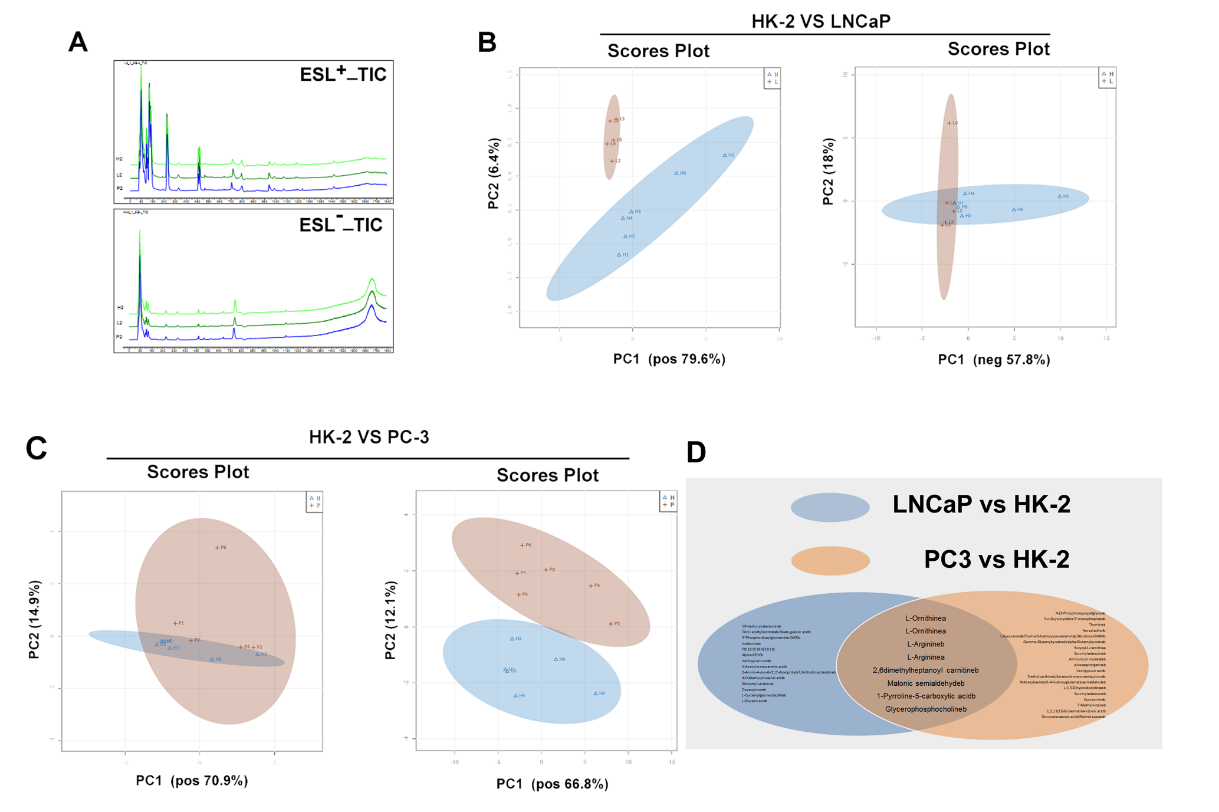


**Figure S6. Quality Control of the Metabonomics, Rlated to Figure 5**

(A) The total ion current chromatogram of samples.

(B) The PCA score plots of LNCaP vs HK-2.

(C) The PCA score plots of PC-3 vs HK-2.

(D) The venn diagram comparing the 21 differentially expressed metabolites in LNCaP vs. the 26 in PC3.


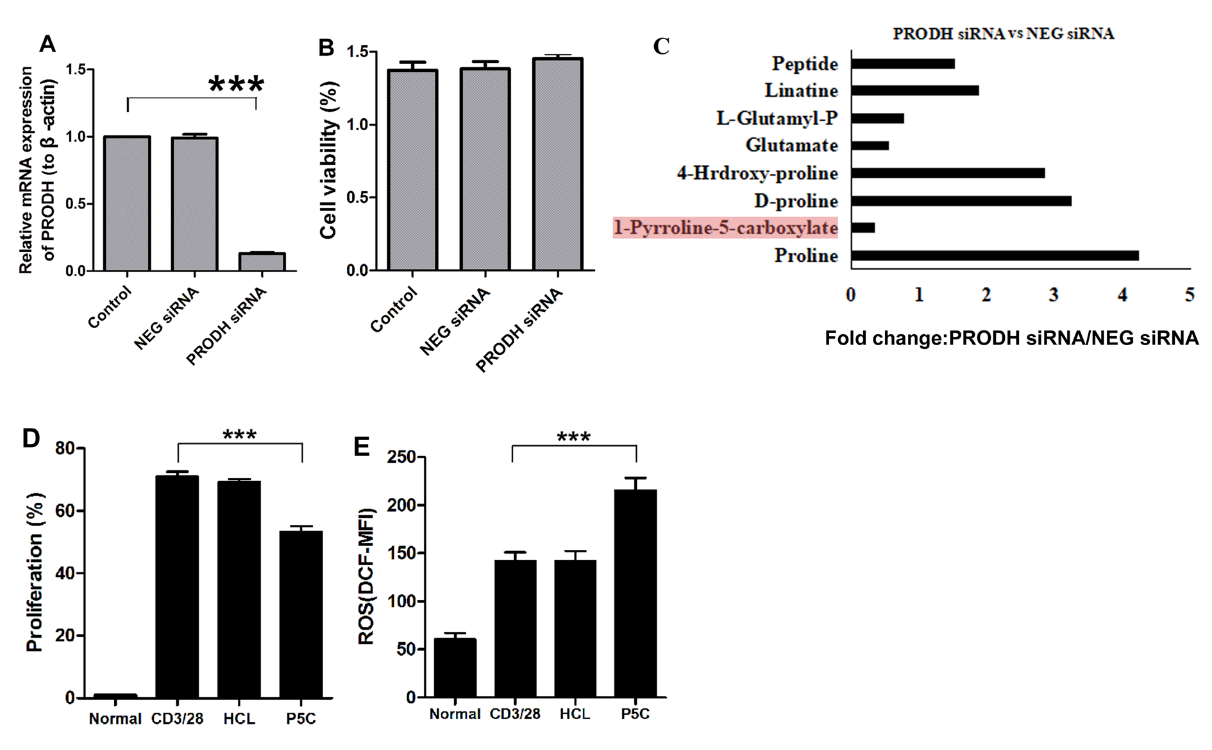


**Figure S7. The Efficiency of PRODH Knockdown and the Effect to Cell Number, and the Effect of Additional P5C on Human CD3^+^ T Cells, Related to Figure 6**

(A) The expression of PRODH in LNCaP after transfection by qPCR.

(B) The cell number of LNCaP after transfection by CCK-8 assay.

(C) The content of P5C (red marked) in PRODH knockdown cells cultured media obviously less than the content in control cells.

(D) Human CD3^+^ T cells were activated for 72h and treated with additional P5C. Shown is the percentage of cell proliferation by flow cytometry. One representative experiment out of three performed.

(E) Human CD3^+^ T cells were activated for 72h and treated with additional P5C. Shown is the levels of ROS by flow cytometry.

All experiments were repeated at least three times. Error bars are SEM of biological replicates and ****P<*0.001.


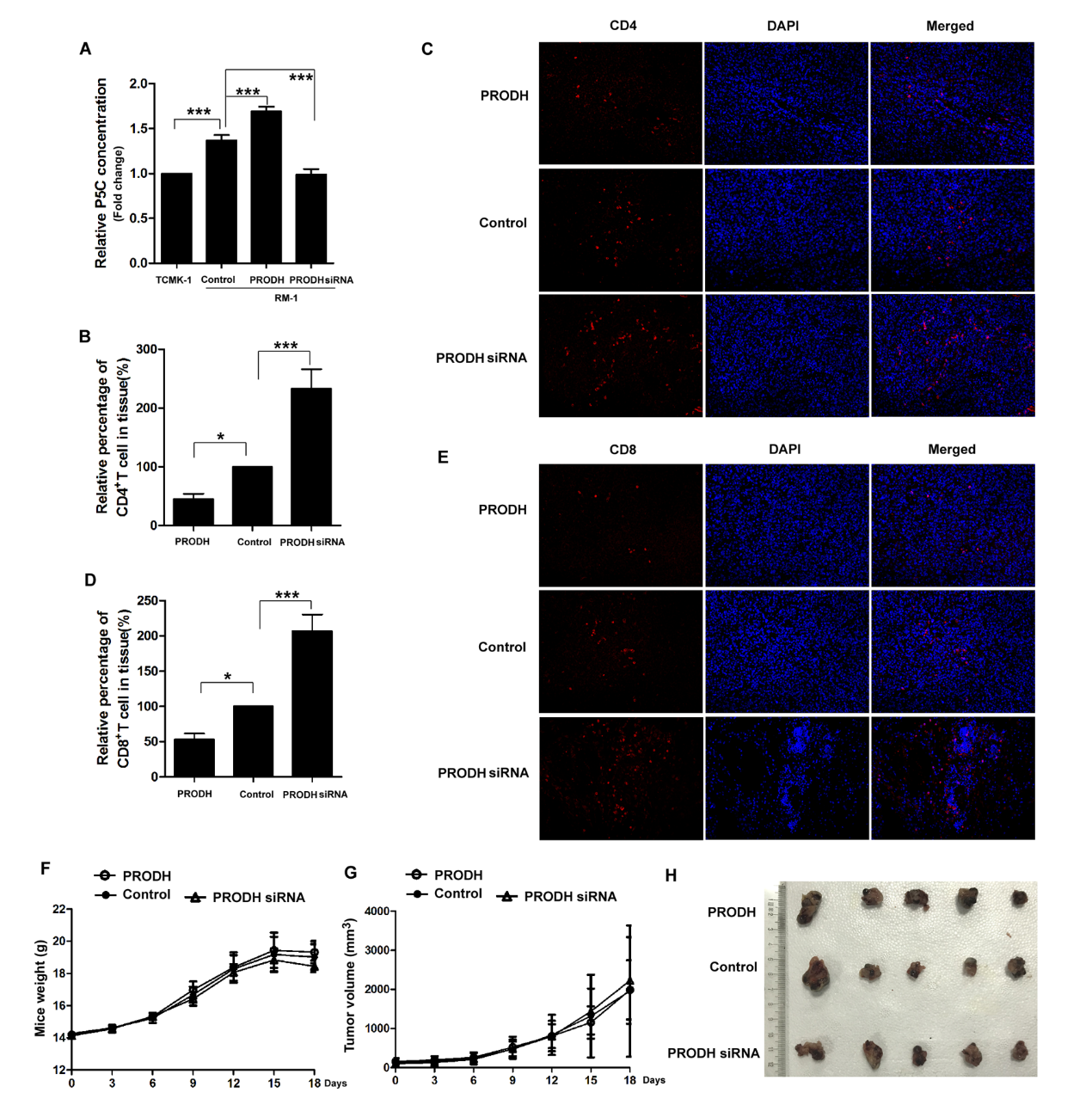


**Figure S8. The Change of PRODH Expression Affect CD4^+^ and CD8^+^ T cells Infiltration in vivo Which Have no Influence on Nude Mice Xenograft**

(A) The relative concentration of P5C in different media of four kinds of cells, including TCMK-1, RM-1, and PRODH overexpression/knockdown RM-1.

(B) Columns showed the quantitative statistics of the infiltration of CD4^+^ T cells.

(C) The infiltration of CD4^+^ T cells in tumor tissue detected by IF. The red light marked CD4^+^ T cells.

(D) Columns showed the quantitative statistics of the infiltration of CD8^+^ T cells.

(E) The infiltration of CD8^+^ T cells in tumor tissue detected by IF. The red light marked CD8^+^ T cells.

(F) Mean of body weight of mice measured at the indicated number of days on nude mice.

(G) Mean of tumor volume measured at the indicated number of days on nude mice.

(H) The picture of tumors on nude mice after harvesting.

Error bars are SEM of biological replicates and **p* <0.05; ****P<*0.001.

**Supplementary Tables**

Table Ⅰ The clinical information on the patients

|  |
| --- |

Patients BPH PCa

| 50-60 2 6 |
| --- |

Age 60-70 5 7

70-80 5 9

> 80 3 3

T1 5

T2 8

Stage T3 7

T4 5

| Total 15 25 |
| --- |
